# Gold Coast criteria for ALS diagnosis: individual participant data meta-analysis

**DOI:** 10.1007/s00415-026-14046-y

**Published:** 2026-08-19

**Authors:** Nipun Shrestha, Zachary Munn, Aicee Calma, Nathan Pavey, Yukiko Tsuji, Parvathi Menon, Alessandro Padovani, Barbara Risi, Lucia Ferullo, Massimiliano Filosto, Gordon Jewett, Kazumoto Shibuya, Ryo Otani, Toshio Shimizu, Satoshi Kuwabara, Yu-ichi Noto, Takamasa Kitaoji, Birger Johnsen, Dongchao Shen, Liying Cui, Leonard H. van den Berg, Ruben P. A. van Eijk, Philip van Damme, Matthew C. Kiernan, Lynne Giles, Steve Vucic

**Affiliations:** 1 Health Evidence Synthesis Recommendation and Impact, School of Public Health, Adelaide University, Adelaide, Australia; 2 Brain and Nerve Research Centre, The University of Sydney, Sydney, Australia; 3 Department of Neurology, Graduate School of Medical Science, Kyoto Prefectural University of Medicine, Kyoto, Japan; 4 Unit of Neurology, ASST Spedali Civili, Brescia, Italy; 5 Department of Clinical and Experimental Sciences, University of Brescia, Brescia, Italy; 6 Department of Molecular and Translational Medicine, University of Brescia, Brescia, Italy; 7 NeMO-Brescia Clinical Center for Neuromuscular Diseases, Brescia, Italy; 8 Department of Clinical Neurosciences, Cumming School of Medicine, University of Calgary, Calgary, Alberta Canada; 9 Hotchkiss Brain Institute, Cumming School of Medicine, University of Calgary, Calgary, AB Canada; 10 Department of Neurology, Graduate School of Medicine, Chiba University, Chiba, Japan; 11 Department of Neurology, Tokyo Metropolitan Neurological Hospital, Tokyo, Japan; 12 Kyoto Prefectural University of Medicine, Kyoto, Japan; 13 Department of Clinical Neurophysiology, Aarhus University Hospital, Aarhus, Denmark; 14 Department of Neurology, Peking Union Medical College Hospital, Beijing, 100730 China; 15 Neuroscience Center, Chinese Academy of Medical Sciences and Peking Union Medical College, Beijing, 100730 China; 16 Department of Neurology, UMC Utrecht Brain Center Rudolf Magnus, Utrecht, The Netherlands; 17 Biostatistics and Research Support, Julius Center for Health Sciences and Primary Care, University Medical Center Utrecht, Utrecht, The Netherlands; 18 Department of Neurology, University Hospitals Leuven, Leuven, Belgium; 19 Neuroscience Research Australia, UNSW Sydney, Randwick, Sydney Australia; 20 School of Public Health, Adelaide University, Adelaide, Australia; 21 Northcott Chair of Neurology Concord Clinical School, Concord Hospital, Clinical School Building 20, Concord West, Sydney, NSW 2139 Australia

**Keywords:** Amyotrophic lateral sclerosis, Awaji and revised El Escorila criteria, Gold Coast criteria

## Abstract

**Background:**

To evaluate the diagnostic accuracy of the Gold Coast criteria (GCC) and compare their performance with the revised El Escorial (rEEC) and Awaji criteria in patients with suspected amyotrophic lateral sclerosis (ALS).

**Methods:**

Embase, MEDLINE, and Scopus were searched for English-language studies published between January 1, 2020, and August 11, 2025. Eligible studies assessed the diagnostic accuracy of GCC compared with rEEC and Awaji criteria in suspected ALS. Authors were invited to contribute individual participant data. Data were checked, harmonised, and recoded. A one-stage individual-participant data meta-analysis, adjusted for age and sex, was performed. Diagnostic performance was assessed using pooled sensitivity, specificity, and area under the receiver operating characteristic curve. Risk of bias was assessed using QUADAS-2 and QUADAS-C, and certainty of evidence using GRADE for diagnostic test accuracy. The study was registered with PROSPERO, CRD420251123597.

**Results:**

Individual participant data were available for 3007 participants from five international studies. GCC demonstrated higher sensitivity than rEEC and Awaji criteria:

0.96 (95% confidence interval [CI] 0.93–0.98) versus 0.87 (95% CI 0.78–0.92) and 0.87 (95% CI 0.78–0.93), respectively. Certainty of evidence for sensitivity was moderate at pre-test probabilities of 50% and 75%, and low at 25%. Specificity was numerically lower for GCC at 0.68 (95% CI 0.53–0.81) compared with rEEC at 0.73 (95% CI 0.59–0.83) and the Awaji criteria at 0.72 (95% CI 0.57–0.83). The certainty of evidence for specificity was rated as very low across all assessed pre-test probabilities (25%, 50%, and 75%).

**Conclusions:**

GCC provide a sensitive framework for suspected ALS and may support earlier diagnosis in specialist settings. Specificity was imprecise and heterogeneous, supporting use with mimic exclusion and longitudinal reassessment.

**Supplementary Information:**

The online version contains supplementary material available at https://doi.org/10.1007/s00415-026-14046-y.

## Introduction

Amyotrophic lateral sclerosis (ALS) is a rapidly progressing, fatal, multisystem neurodegenerative disorder that predominantly affects the motor system but is also associated with cognitive, behavioural, and other non-motor manifestations [1]. Diagnosis is challenging, requiring evidence of upper (UMN) and lower motor neuron (LMN) involvement within the same region, or LMN involvement across two or more regions, in the setting of a progressive motor syndrome [2–4]. Given its clinical heterogeneity and lack of pathognomonic tests, diagnostic criteria incorporating clinical and neurophysiological features have been developed to support patient management and trial recruitment [5, 6].

The original El Escorial criteria defined four levels of diagnostic certainty: definite, probable, possible, and suspected ALS [7]. The revised El Escorial (Airlie House, rEEC) criteria aimed to improve sensitivity by introducing a “laboratory-supported probable ALS” category [8]. The Awaji criteria addressed limitations of relying on ongoing denervation changes, which may be absent in affected muscles [9], by incorporating fasciculations with chronic neurogenic change as evidence of LMN dysfunction and removing the “laboratory-supported probable ALS” category [10]. The categories of definite, probable, and possible ALS were retained, with neurophysiological measures permitted across all levels of diagnostic uncertainty. These diagnostic criteria are limited by their complexity, resulting in low inter‐rater reliability [11]. They may not reflect disease stage, with up to 22% of patients dying while classified as “possible ALS” [12]. The Gold Coast diagnostic criteria (GCC) were developed to address these limitations (Box 1) [13]. Cohort studies across Europe, North America, and Asia–Pacific have consistently shown higher sensitivity of GCC across onset site, disease duration, and functional disability, particularly for LMN-predominant phenotypes, while appropriately excluding primary lateral sclerosis (PLS) [14–21]. However, specificity remains more variable.

**Box 1:** Gold Coast criteria for the diagnosis of amyotrophic lateral sclerosis

Requirement	Definition
1. Progressive motor impairment	Progressive motor impairment documented by the clinical history or repeated clinical assessment and preceded by a period of normal motor function
2. Upper and lower motor neuron dysfunction	Upper and lower motor neuron dysfunction in at least one body region, with both present in the same region when only one region is involved; or lower motor neuron dysfunction in at least two body regions
3. Exclusion of alternative disease processes	Clinically appropriate investigations excluding other disease processes that could account for the presentation. The investigations should be guided by the clinical presentation and may include nerve conduction studies and needle electromyography, magnetic resonance or other imaging, blood or cerebrospinal fluid studies, and other tests as clinically indicated

The diagnosis of ALS requires all three points. ^**a**^Upper motor neuron dysfunction requires at least one of the following: Increased deep tendon reflexes, including preserved or exaggerated reflexes in a weak and wasted muscle or spread of reflexes to adjacent muscles, Pathological reflexes, such as the Hoffmann, Babinski, crossed adductor, or snout reflex, Velocity-dependent increased muscle tone or spasticity, Slowed or poorly coordinated voluntary movement that is not attributable to lower motor neuron weakness or parkinsonian features. ^**b**^Lower motor neuron dysfunction requires either: Clinical evidence of muscle weakness and muscle wasting; or, Electromyographic evidence of both chronic neurogenic change and ongoing denervation, including fibrillation potentials, positive sharp waves, or fasciculation potentials. Body regions are defined as bulbar, cervical, thoracic, and lumbosacral. For lower motor neuron involvement, a region is considered affected when abnormalities are present in two limb muscles innervated by different roots and peripheral nerves, or in one bulbar or one thoracic muscle.

A recent aggregate-data meta-analysis reported higher pooled sensitivity but lower specificity for GCC [22]. The pooled estimates were derived from studies using different reference standards, potentially limiting comparability across studies. A smaller aggregate-data meta‐analysis, incorporating data from three studies, reported higher pooled sensitivity (0.93, 95% CI 0.88 to 0.96) and specificity (0.84, 95% CI 0.22 to 0.99) for GCC, although the wide confidence interval around specificity indicated substantial imprecision [23]. As such, these findings should be interpreted cautiously because of the small number of included studies and the use of aggregate rather than individual-participant data.

In the absence of reliable pooled estimates comparing the accuracy of different diagnostic criteria for ALS, an individual participant data (IPD) meta-analysis was conducted to compare the diagnostic accuracy of the Gold Coast, Awaji, and rEEC criteria in ALS.

## Methods

This systematic review was registered in PROSPERO (CRD420251123597) and initially planned as an aggregate data meta-analysis. Corresponding authors from seven studies agreed to share individual participant data to address missing information [14–20]. Raw data were obtained from seven studies, of which five met eligibility criteria and were included in an individual participant data meta-analysis [15, 16, 18–20]. Data checking, cleaning, and a predefined statistical analysis plan were completed before analysis. Results were reported in accordance with the Preferred Reporting Items for Systematic Reviews and Meta-Analyses (PRISMA-IPD and PRISMA-DTA) checklists [24, 25].

### Study eligibility

The *inclusion criteria* were as follows: (1) Studies assessing the diagnostic accuracy of the Gold Coast, Awaji, and rEEC criteria in adults (≥ 18 years of age) clinically suspected of having ALS; (2) studies in which participants were assigned a final reference-standard diagnosis of ALS or non-ALS following clinical follow-up, based on expert clinical assessment, disease progression, relevant investigations, and exclusion or identification of an alternative diagnosis; and (3) were cross-sectional or cohort in design. The review-level eligibility criteria did not impose an upper age limit or maximum symptom duration; therefore, all available participants meeting these criteria were eligible for inclusion in the IPD analysis.

The *exclusion criteria* were as follows: (1) Reviews, case reports, editorials, case–control studies with less than 10 participants; (2) Studies in which the index test could not be derived, such that true positives, false positives, true negatives, or false negatives could not be determined against the reference standard; (3) Studies that did not directly compare the diagnostic accuracy of the GCC with the rEEC and/or Awaji criteria were also excluded.

### Reference standard

As no pathognomonic diagnostic test exists for ALS, the reference standard was the final clinical diagnosis assigned in each included study. This was based on expert clinical assessment at follow-up, incorporating the longitudinal pattern of disease progression, neurological examination findings, neurophysiological and other ancillary investigations, and the exclusion or identification of alternative diagnoses. Participants were classified as having ALS when their subsequent clinical course was considered consistent with ALS and as having a non-ALS disorder when an alternative diagnosis was established or when follow-up did not support ALS. The precise reference-standard definition, duration of follow-up, and diagnostic adjudication process used in each study were extracted (Table 1).

**Table 1:** Characteristics of the included studies

Study ID	Country	Time period	Patient Sampling	Patient Characteristics, Setting and Sample size	Index Tests	Reference Standard(s)
Hannaford 2021^4^	Australia	January 2012—October 2020	Prospective consecutive enrolment; retrospective application of index tests	Patients attending two major ALS centres in Sydney, Australia, who were referred with suspected ALS or a neuromuscular disorder (muscle weakness and wasting ≥ 6 months in ≥ 1 body region, with or without sensory symptoms) *n* = 506	Gold Coast, Awaji, and revised El Escorial (rEEC) criteria	Combination of disease progression over 6 months and exclusion of mimicking disorders during follow-up
Shen 2021^8^	China	January 2014- December 2019	Prospective consecutive enrolment; retrospective application of index tests	Patients with suspected ALS attending Peking Union Medical College Hospital and enrolled in a registry n = 1185	Gold Coast, Awaji, and rEEC criteria	Diagnosis at last follow-up (every 6 months until tracheostomy, death, or loss to follow-up) served as the final diagnosis
Otani 2024^6^	Japan	September 2013—June 2020	Prospective consecutive enrolment; retrospective application of index tests	Patients with progressive motor impairment and suspected ALS who underwent systematic neurophysiological testing at an electromyography (EMG) laboratory *n* = 639	Gold Coast, Awaji, and rEEC criteria	Final diagnosis determined by attending physicians based on clinical course, test results, and exclusion of other diseases at last visit
Pugdahl 2021^7^*	Denmark	Oct 2011–Jul 2020	Retrospective sampling of consecutive adults referred for electrodiagnostic testing on suspicion of ALS	Adults aged 20–80 years with ≤ 36 months of symptoms, referred for electrodiagnostic testing, and available for clinical follow-up. EMG included motor unit potentials (MUP) analysis in ≥ 2 muscles in cervical and lumbosacral regions and one bulbar muscle: nerve conduction studies in ≥ 2 motor and ≥ 2 sensory nerves, *n* = 442	Gold Coast, Awaji, and rEEC criteria	Reference diagnosis confirmed by disease progression, assisted ventilation, or death. Non-ALS: alternative diagnoses or no progression ≥ 12 months
Ferullo 2024^3^	Italy	June 2012—May 2023	Prospective enrolment	Patients and controls recruited at the Unit of Neurology and NeMO-Brescia Clinical Center for Neuromuscular Diseases, Italy (June 2012–May 2023) *n* = 235	Gold Coast, Awaji, and rEEC criteria	ALS diagnosis confirmed by progressive disease course consistent with clinical criteria; non-ALS: other diagnoses or no progression ≥ 12 months

^#^*rEEC* revised El escorial criteria, *ALS* amyotrophic lateral sclerosis, *EMG* electromyography, *MUP* motor unit action potential

^*^The IPD provided for Pugdahl et al. included participants aged > 80 years and/or with symptom duration > 36 months who met the review-level eligibility criteria but were outside the eligibility criteria applied in the original publication. A sensitivity analysis applying the original published criteria is presented in Supplementary Table 4

### Database searches and study selection

The GCC was reported in 2020 and the first study assessing diagnostic utility was published in 2021[16]. A systematic literature search was conducted for studies published in the English language from January 1, 2020, to August 11, 2025. Embase, MEDLINE, and Scopus were searched. The search strategies are presented in Supplementary 1*.* Search results were uploaded to Covidence; duplicates were removed, and the titles and abstracts of the eligible studies were then reviewed independently by three authors (NP, AC, PM) against the eligibility criteria. Three authors (NP, AC and PM) independently reviewed selected full-text studies. Differences were resolved by consensus, with a fourth reviewer consulted if necessary.

### Outcomes

The primary outcome was the comparative diagnostic accuracy of the GCC, Awaji, and rEEC criteria in patients clinically suspected of having ALS. Sensitivity was estimated among participants assigned a final reference-standard diagnosis of ALS, whereas specificity was estimated among participants assigned a final diagnosis of non-ALS. A prespecified subgroup analysis assessed diagnostic performance after excluding participants classified as definite ALS under the revised El Escorial or Awaji criteria.

### Data request, synthesis and analysis

De-identified IPD were obtained for age at assessment, sex, disease duration, site of onset, ALS Functional Rating Scale scores, GCC, Awaji, and rEEC criteria ratings, and final diagnosis. Data were checked for invalid, inconsistent, out-of-range, and missing values and cross-referenced against published reports. Discrepancies were resolved through follow-up with corresponding authors. In accordance with the review-level eligibility criteria, participants from Pugdahl and colleagues aged > 80 years and/or with symptom duration > 36 months were retained in the primary analysis, although they were outside the eligibility criteria applied in the original publication. Participants with primary lateral sclerosis (PLS) were re-coded as non-ALS to ensure consistency across studies.

### Data analysis

A one-stage meta-analysis was carried out through fitting a binomial generalised linear mixed-effects model with a logit link to jointly estimate sensitivity and specificity, including study-level random intercepts to account for between-study heterogeneity and correlation between sensitivity and specificity [26, 27]. The model was initially fit without covariates and subsequently adjusted for mean-centered age and sex. Only age and sex were adjusted for in the model because of the high proportion of missing data for other clinically relevant covariates, including site of onset and disease duration. Between-study variances (τ^2^) for logit-sensitivity, logit-specificity, and their covariance, were extracted from each fitted mixed model. The 95% confidence intervals (CI) were computed using the Wald approximation on the logit scale.

In the primary analysis, patients diagnosed with PLS were recoded as non-ALS in Hannaford et al. [16] consistent with the approach adopted by the other included studies (Supplementary Table 1). In pre-specified sensitivity analyses, patients diagnosed with PLS were excluded because the Awaji and rEEC criteria were not designed to distinguish PLS from ALS. A further sensitivity analysis restricted the Pugdahl et al. cohort to the eligibility criteria reported in the original publication, namely age 20–80 years and symptom duration ≤ 36 months. Additionally, a leave-one-out analysis was undertaken to evaluate the robustness of pooled estimates and determine the extent to which individual studies contributed to between-study heterogeneity. Subgroup analyses were also planned to assess the performance of GCC in borderline ALS cases by excluding participants with “definite” ALS, as defined by the rEEC and Awaji criteria, in which diagnostic uncertainty is greatest, and misclassification is most likely to occur. We visually inspected forest plots and summary receiver operating characteristic (SROC) curves to assess heterogeneity across all analyses based on the one-stage model.

A two-stage meta-analysis was also conducted, using a bivariate generalised linear mixed model to compare estimates with those obtained from the one-stage meta-analysis without covariates. From the two-stage model, the coupled forest plot and ROC curves were generated using RevMan web [28]. Overall diagnostic accuracy for each criterion was summarised using the area under the curve (AUC) of the summary ROC curve. The mean AUC and corresponding 95% CIs were estimated from the percentile distribution of bootstrapped AUC values. An AUC of 0.90–1.00 was considered excellent; 0.80–0.89, good; 0.70–0.79, fair and < 0.70, poor [27]. Funnel plots or regression tests were not used for assessing study bias because of their limitations in diagnostic test accuracy studies [26]. All analyses were performed in R software (version 4.2) using the lme4 and emmeans packages [29].

### Risk of bias assessment

Risk of bias was assessed using the Quality Assessment of Diagnostic Accuracy Studies; QUADAS-2 [30] and QUADAS-C [31] tools in the included studies using publication and information provided by study authors. All the studies were assessed independently by two reviewers (NS and ZM), and any discrepancies were resolved through discussion or, if necessary, by consulting a third reviewer.

### Certainty of evidence assessment

We assessed certainty of evidence for GCC compared with Awaji and rEEC criteria using the GRADE approach for diagnostic studies and prepared summary of findings tables with GRADEpro GDT (https://gdt.gradepro.org/app). Certainty was rated across risk of bias, indirectness, inconsistency, imprecision, and publication bias. Evidence certainty was evaluated at pre-test probabilities of 25%, 50%, and 75%, given absence of prevalence data in patients referred to specialist ALS clinics. The GRADE assessment was informed by the riluzole evidence profile for mortality and adverse events, and decision thresholds from the Australian motor neurone disease guidelines [32] (Supplementary 2). Downstream clinical consequences were considered descriptively, including the potential benefits of earlier diagnosis and the potential harms of false-positive classification. Where treatment effects from riluzole studies were considered, these were interpreted as indirect contextual evidence rather than as direct estimates of benefit attributable to earlier diagnosis under GCC.

## Results

### Study selection and characteristics

The study selection process is described in the PRISMA flow chart (Supplementary Fig. 1). During the initial search, 37 studies were identified from MEDLINE, Embase, and Scopus databases. Following title and abstract screening, 13 studies underwent full-text assessment. [14–21, 33–37]. Of these, eight studies were excluded, while five [15, 16, 18–20] met the inclusion criteria and were included in the IPD meta-analysis. (Table 1). Regarding eight excluded studies, three were excluded due to a wrong index test [21, 33, 34], two for an out-of-scope design [36, 37] and three did not have a non-ALS control group [14, 17, 35] (Supplementary Table 2). IPD was sought and received for all five eligible studies [15, 16, 18–20].

For three studies [15, 16, 18], ALS diagnosis was based on good clinical practice, with disease progression used as reference standard, requiring exclusion of mimic disorders. Shen et al. [20] used the clinical diagnosis recorded at last follow-up as reference standard. In contrast, Pugdahl et al. [19] defined ALS based on disease progression, while patients with alternative diagnoses and no disease progression for at least 12 months were classified as non-ALS.

In total, 2443 ALS and 564 pathological neuromuscular non-ALS patients from five studies were assessed. Demographic and clinical characteristics of all participants are summarised in Supplementary Table 3. Limb-onset disease was present in 72% of ALS patients for whom this information was available, whereas 88% of non-ALS patients had limb-onset. The mean age at assessment of ALS patients was 59.3 (SD 12.6) years, with a mean disease duration of 16.8 (SD 17.3) months. Moderate functional disability was evident among ALS patients (Supplementary Table 3) as indicated by a median Revised ALS Functional Rating Scale (ALSFRS-R) score of 40 (IQR 35- 44).

### Diagnostic accuracy

#### Pooled Analysis

**Fig. 1:**
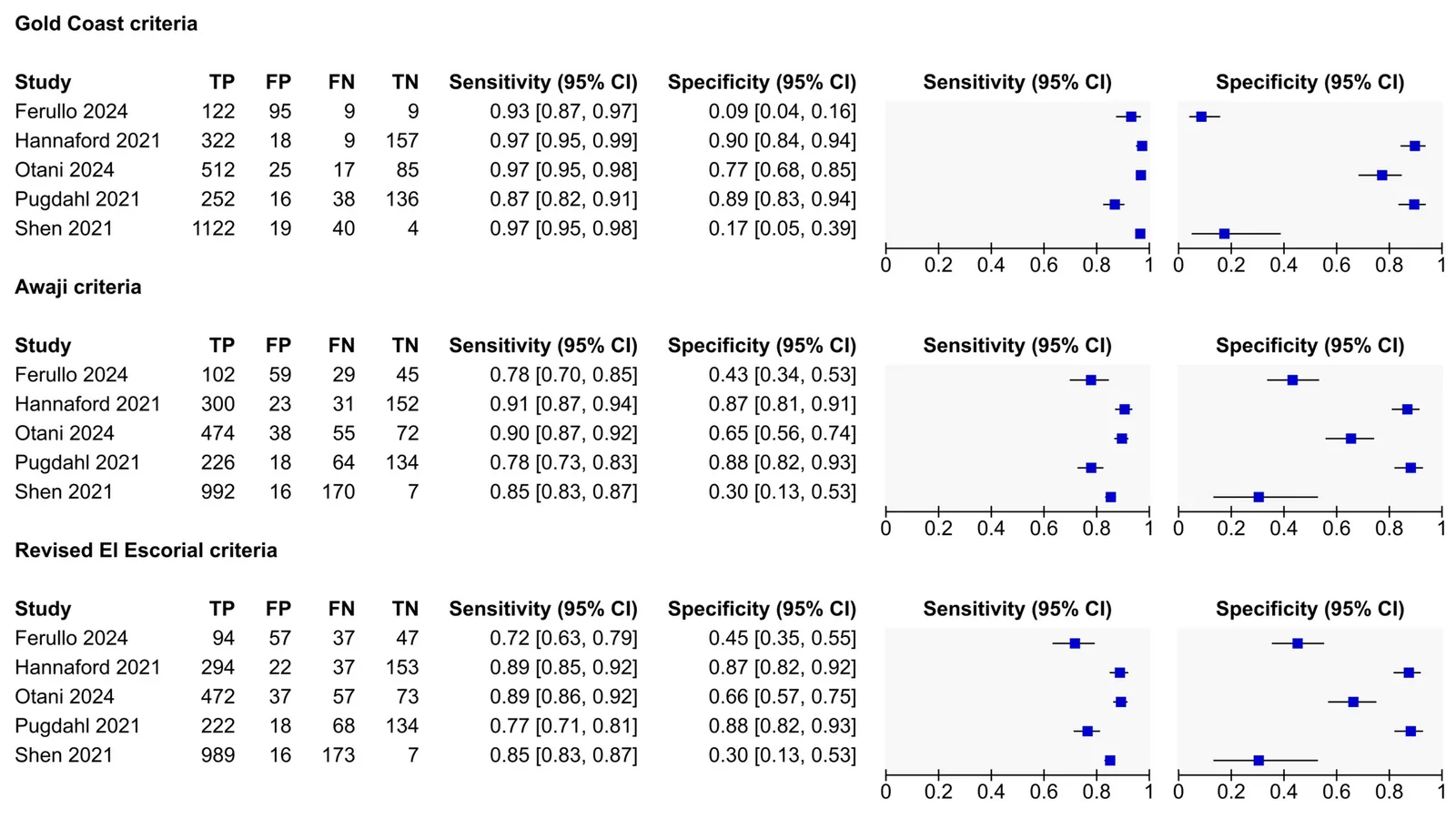
Forest plots depicting the sensitivity and specificity of the Gold Coast, Awaji, and revised El Escorial criteria

**Fig. 2:**
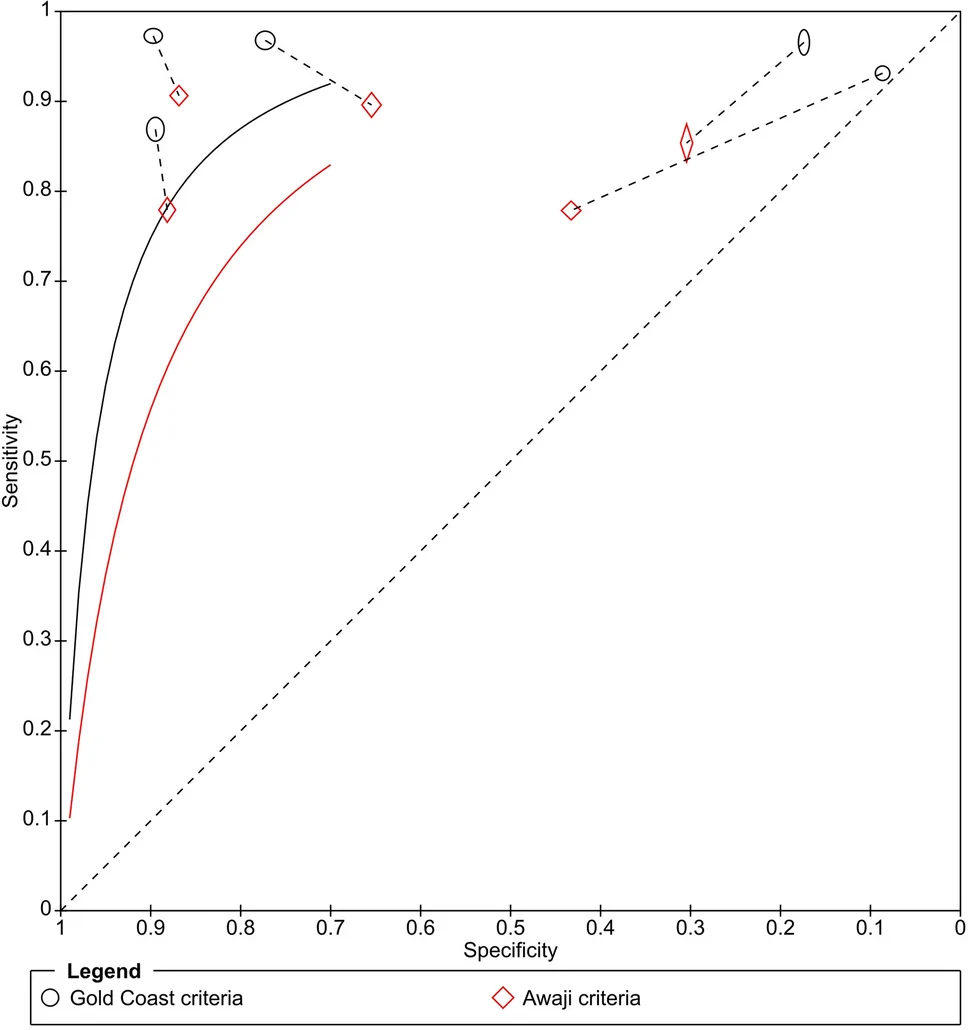
Summary receiver operating characteristic (SROC) curves for Gold Coast and Awaji criteria. SROC curves showing the diagnostic performance of Gold Coast and the Awaji criteria across the included studies. Open black circles represent individual study estimates for the Gold Coast criteria, and open red diamonds represent individual study estimates for the Awaji criteria. Solid curves show the fitted SROC curves for each diagnostic criterion. Dashed connecting lines indicate paired estimates from the same study or cohort where both criteria were evaluated. The diagonal dashed line represents the line of no diagnostic discrimination. Sensitivity is plotted against specificity, with specificity displayed on a reversed x-axis

**Fig. 3:**
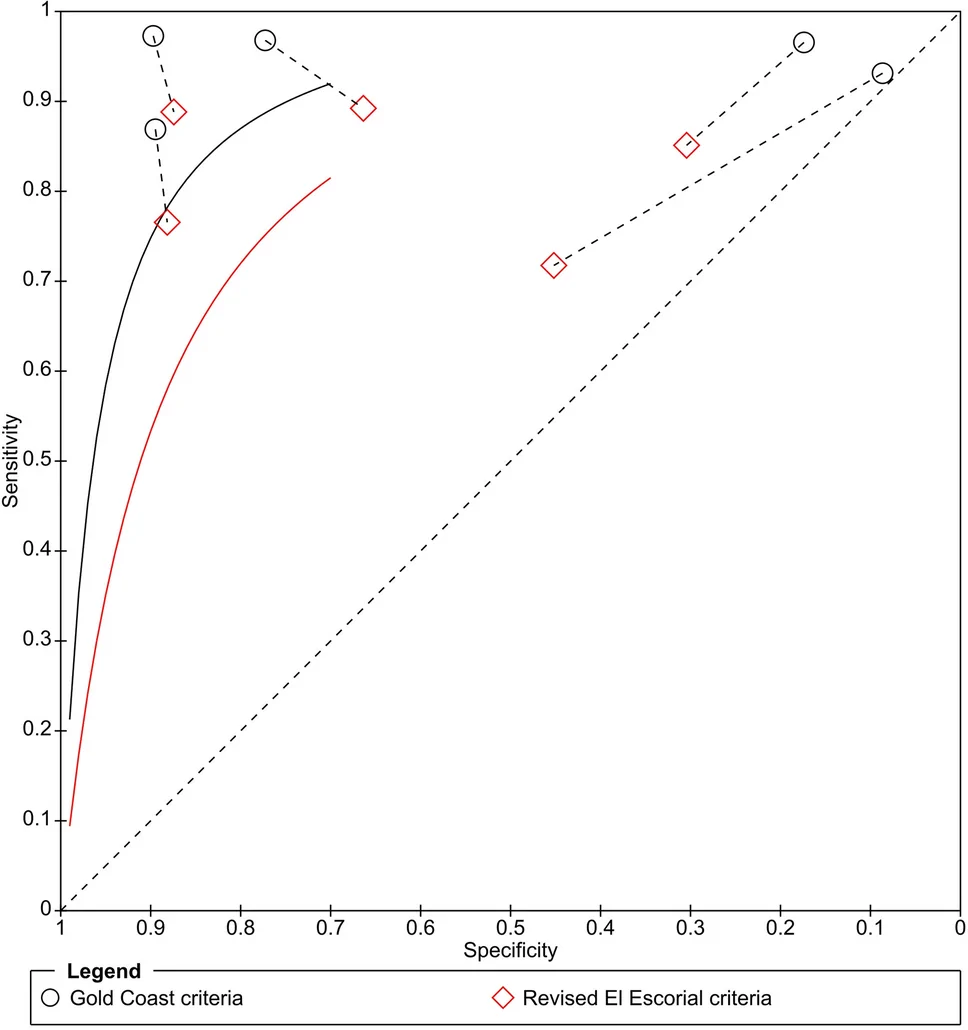
Summary receiver operating characteristic curves (SROC) comparing Gold Coast and revised El Escorial criteria. SROC curves showing the diagnostic performance of the Gold Coast criteria and revised El Escorial criteria across included studies. Open black circles represent individual study estimates for the Gold Coast criteria, while open red diamonds represent individual study estimates for the revised El Escorial criteria. Solid curves indicate the fitted SROC curves for each diagnostic criterion. Dashed connecting lines indicate paired estimates from the same study or cohort where both criteria were evaluated. The diagonal dashed line represents no diagnostic discrimination. Sensitivity is plotted against specificity, with specificity displayed on a reversed x-axis

The pooled estimate of sensitivity for GCC was 0.96 (95% CI 0.93–0.98), while specificity was 0.68 (95% CI 0.53–0.81), derived from the model including covariates (Fig. 1, Table 2). For Awaji criteria, sensitivity was 0.87 (95% CI 0.78–0.93) and specificity 0.72 (95% CI 0.57–0.83), closely mirroring the rEEC criteria, which showed a sensitivity of 0.87 (95% CI 0.78–0.92) and specificity of 0.73 (95% CI 0.59–0.83) [Fig. 1, Table 2]. These estimates were similar to those obtained from the one-stage model without covariates. There was low to moderate heterogeneity for sensitivity, as indicated by relatively narrow prediction intervals, and substantial heterogeneity for specificity, as reflected by considerably wider prediction intervals. A leave-one-out analysis did not identify any individual study that substantially influenced pooled sensitivity or specificity estimates. However, despite the substantial heterogeneity observed for specificity, the small number of included studies limited the ability of this analysis to determine the sources of heterogeneity (Supplementary Table 4). The AUC was excellent for GCC (0.94, 95% CI 0.84–0.97), and very good for Awaji and rEEC criteria [Table 2, Figs. 2, 3].

**Table 2:** Study results

Criteria	Number of studies	Number of participants	Sensitivity			Specificity			Area under curve
			Pooled estimate	τ^2^	Prediction interval	Pooled estimate	τ^2^	Prediction interval	
*One stage model without covariates*									
Gold Coast	5	3007	0.96 (95% CI 0.93 to 0.98)	0.60	0.84 to 0.98	0.68 (95% CI 0.53 to 0.81)	1.91	0.01 to 0.98	
Awaji			0.88 (95% CI 0.79 to 0.93)	0.39	0.71 to 0.93	0.72 (95% CI 0.57 to 0.84)	1.10	0.04 to 0.84	
Revised El Escorial			0.87 (95% CI 0.78 to 0.92)	0.44	0.66 to 0.93	0.73 (95% CI 0.59 to 0.83)	1.43	0.02 to 0.89	
*One stage model with covariates*									
Gold Coast	5	3007	0.96 (95% CI 0.93 to 0.98)	0.58	0.86 to 0.99	0.68 (95% CI 0.53 to 0.81)	1.89	0.01 to 0.98	
Awaji			0.87 (95% CI 0.78 to 0.93)	0.39	0.72 to 0.93	0.72 (95% CI 0.57 to 0.83)	1.05	0.05 to 0.83	
Revised El Escorial			0.87 (95% CI 0.78 to 0.92)	0.44	0.67 to 0.93	0.73 (95% CI 0.59 to 0.83)	1.41	0.01 to 0.88	
Sensitivity analysis- One stage model^*^									
Gold Coast	5	2982	0.96 (95% CI 0.93 to 0.98)	0.58	0.86 to 0.99	0.67 (95% CI 0.51 to 0.80)	1.86	0.01 to 0.98	
Awaji			0.88 (95% CI 0.79 to 0.94)	0.40	0.72 to 0.94	0.76 (95% CI 0.61 to 0.86)	1.39	0.02 to 0.88	
Revised El Escorial			0.87 (95% CI 0.78 to 0.92)	0.44	0.67 to 0.93	0.76 (95% CI 0.63 to 0.86)	1.41	0.02 to 0.88	
*Subgroup analysis- One stage model*^*#*^*: Gold Coast vs. Awaji*									
Gold Coast	5	2283	0.95 (95% CI 0.90 to 0.97)	0.40	0.86 to 0.97	0.69 (95% CI 0.53 to 0.82)	1.87	0.01 to 0.97	
Awaji			0.82 (95% CI 0.70 to 0.90)	0.16	0.73 to 0.85	0.73 (95% CI 0.58 to 0.85)	1.01	0.05 to 0.80	
*Subgroup analysis- One stage model*^*#*^*: Gold Coast vs. revised El Escorial*									
Gold Coast	5	2480	0.95 (95% CI 0.91 to 0.97)	0.43	0.86 to 0.98	0.68 (95% CI 0.53 to 0.80)	1.90	0.01 to 0.98	
Revised El Escorial			0.82 (95% CI 0.71 to 0.90)	0.27	0.68 to 0.88	0.73 (95% CI 0.59 to 0.84)	1.04	0.04 to 0.81	
*Two stage (Bivariate glmm) model*									
Gold Coast	5	3007	0.95 (95% CI 0.92 to 0.97)			0.61 (95% CI 0.33 to 0.84)			0.94 (95% CI 0.84 to 0.97)
Awaji			0.85 (95% CI 0.77 to 0.89)			0.67 (95% CI 0.38 to 0.86)			0.86 (95% CI 0.76 to 0.91)
Revised El Escorial	5		0.84 (95% CI 0.77 to 0.89)			0.68 (95% CI 0.39 to 0.87)			0.85 (95% CI 0.72 to 0.91)

^*^Sensitivity analysis after excluding primary lateral sclerosis patients from the database

^#^Subgroup analysis with only borderline ALS cases (Probable or possible ALS). *ALS* amyotrophic lateral sclerosis

τ^2^, estimated between-study variance on the logit scale; CI, confidence interval; GLMM, generalised linear mixed model

For a pretest probability of 25%, the certainty of the evidence for GCC sensitivity was judged to be low, whereas the certainty of the evidence for specificity was very low, compared with the Awaji and rEEC criteria. At pretest probabilities of 50% and 75%, certainty of evidence for sensitivity was judged to be moderate, whereas certainty for specificity was very low (Supplementary Table 5, Supplementary Table 6). The downstream consequences presented in the GRADE summary should be interpreted as illustrative estimates, as the clinical benefit of earlier diagnosis depends on the timing of treatment initiation, access to multidisciplinary care, and the accuracy with which mimic disorders are excluded.

In a prespecified sensitivity analysis excluding PLS patients, pooled sensitivity estimates remained consistent with those from the full one-stage model. Gold Coast criteria specificity remained unchanged at 0.67, whereas rEEC and Awaji specificity increased from 0.72 to 0.76 (Table 2). This suggests that inclusion of PLS patients as non-ALS did not account for the numerically lower specificity estimate for GCC, consistent with the design of the GCC, which explicitly distinguishes ALS from PLS [13]. Additionally, restricting the Pugdahl et al. cohort to participants aged 20–80 years with symptom duration ≤ 36 months did not materially alter the pooled sensitivity or specificity estimates (Supplementary Table 4).

Subgroup analyses excluding participants with a “definite” diagnosis of ALS were also performed (Supplementary Fig. 2, Supplementary Fig. 3). Pooled estimates of sensitivity and specificity were similar to those derived from the overall one-stage model for GCC, while the sensitivity was slightly lower for Awaji and rEEC (Table 2). Separately, pooled sensitivity and specificity estimates derived from two stage models for the three criteria were comparable to those derived from the one-stage model (Table 2). Subgroup analyses by site did not converge and therefore are not presented.

### Risk of bias assessments

Using QUADAS-2, patient selection was judged as low risk in four studies as participants were enrolled consecutively [15, 16, 18, 19]. Shen et al. [20] was judged as unclear risk in this domain because of uncertainty regarding the selection and classification of ALS mimic cases. For the index test domain, Ferullo et al. [15] was judged as high risk due to a lack of assessor blinding to the final diagnosis when applying the diagnostic criteria, whereas Otani et al. [18]; was judged as unclear risk because assessor blinding was not reported. Remaining included studies were judged as low risk [16, 19]. All studies were judged as low risk of bias in the QUADAS-2 reference-standard domain because the formal index-criteria classification was not used directly to assign the final diagnosis. This judgement reflects the QUADAS-2 signaling questions and does not imply that the clinical reference standard was pathognomonic or free from uncertainty. The clinical reference standards varied between studies, and this heterogeneity is considered further in the discussion. In the flow and timing domain, Pugdahl et al. [19] and Shen et al.[20] were judged as unclear risk, while the remaining studies were judged as low risk. Applicability concerns were low across all QUADAS-2 domains (Supplementary Fig. 4, Supplementary 3, 4, 5).

Using QUADAS-C, patient selection was judged as unclear risk in Shen et al. [20] with the remaining studies judged as low risk. In the index test domain, Ferullo et al. [15] was judged as high risk and Otani et al. [18] as unclear risk; all other studies were judged as low risk. All studies were judged as low risk in the reference standard domain. In the flow and timing domain, three studies were judged as low risk[15, 16, 18], while two studies Pugdahl et al .[19] and Shen et al. [20] were judged as unclear risk. Overall, comparative risk of bias was high for one study [15], unclear for three[18–20] and low for one study [16].

## Discussion

Utilising individual patient data from 3007 participants across five published studies, the present study assessed diagnostic accuracy of GCC and compared its performance with established Awaji and rEEC criteria. Moderate to low certainty evidence supported a higher sensitivity of GCC, and very low certainty of evidence for lower specificity (Supplementary Table 5, Supplementary Table 6). The Gold Coast criteria demonstrated higher sensitivity, identifying more patients with ALS than the Awaji and revised El Escorial criteria, accompanied by numerically lower specificity. Although the area under curve (AUC) for GCC was high, no formal comparison of AUCs was performed and the confidence intervals overlapped. As such, superiority in overall diagnostic discrimination cannot be inferred. Given their simplicity and close alignment with routine clinical practice, GCC supports earlier ALS diagnosis. Although the GCC improve diagnostic sensitivity, their lower specificity underscores the need for careful clinical evaluation, exclusion of mimic disorders, and longitudinal reassessment, particularly in atypical cases. In therapeutic trials, additional safeguards, including central diagnostic review or biomarker-supported confirmation, may be required to minimise diagnostic misclassification and preserve trial validity.

The present IPD meta-analysis demonstrated that across all analytical approaches, GCC had higher sensitivity compared to Awaji and rEEC criteria, with pooled sensitivity estimates of 0.96 and narrow prediction intervals, indicating minimal between-study heterogeneity (Table 2). The narrow CIs and stable estimates across one-stage, covariate-adjusted, sensitivity, subgroup, and two-stage bivariate models support a moderate level of certainty. The certainty of evidence for sensitivity was rated as moderate due to consistent, precise, and robust findings across multiple IPD modelling approaches, with downgrading driven by residual risk of bias and inherent limitations of the clinical reference standard.

In contrast, specificity estimates for GCC were lower and more variable, with wide CIs and wide prediction intervals, indicating substantial uncertainty and heterogeneity across clinical settings. This variability likely reflects differences in study populations, the spectrum of neuromuscular mimicking disorders, study settings, clinician expertise in applying diagnostic criteria, and variability in reference standards. Ferullo and colleagues [15] reported substantially lower specificity for all three criteria, suggesting that cohort-specific factors, rather than a weakness specific to the GCC, may explain the observed reduction in specificity. This study was also rated at high risk of bias in the QUADAS-2 index-test domain because of the absence of assessor blinding to the final diagnosis, which may have contributed to false-positive classification. Similarly, in Shen and colleagues [20] only 23 of 1,185 participants were classified as non-ALS, including four true negatives and 19 false positives. Thus, despite the large overall cohort, its specificity estimate was based on a very small and markedly imbalanced non-ALS sample and should be interpreted cautiously. Leave-one-out analyses excluding Ferullo and Shen and colleagues in turn did not materially alter the pooled specificity estimate or between-study variance (Supplementary Table 4). However, with only five included studies, these analyses had limited capacity to identify influential studies or explain the sources of heterogeneity. Accordingly, the pooled specificity should be interpreted as a setting-dependent summary rather than a universally applicable estimate, with greater emphasis placed on the summary ROC curve and prediction interval.

Lower specificity may further reflect challenges in reliably differentiating mimicking disorders from ALS across clinical settings. The Gold Coast criteria explicitly require investigations to exclude other disease processes and indicate that appropriate investigations should depend on the clinical presentation, potentially including nerve conduction studies and needle electromyography, imaging, blood or cerebrospinal fluid studies, and other clinically indicated modalities [13]. The criteria, however, do not mandate a single standardised investigation pathway. Variation between clinical settings in the selection, extent, and interpretation of investigations used to exclude mimic disorders may therefore contribute to heterogeneity in specificity estimates. The Gold Coast criteria should be applied within an expert clinical framework incorporating clinically appropriate investigations and longitudinal reassessment when diagnostic uncertainty persists, particularly in atypical or lower motor neuron-predominant presentations. Consequently, the certainty of evidence for specificity was rated as very low. Importantly, diagnostic uncertainty is not unique to GCC, with substantial inter-rater variation reported even among experts applying the rEEC and Awaji criteria, likely reflecting their greater complexity [11]. The simpler binary structure of the GCC may reduce interpretive complexity and potentially improve agreement between clinicians. However, the inter-rater reliability of the GCC has not been directly evaluated, and any advantage in this respect remains inferential and requires formal investigation.

The previous IPD meta-analysis reported sensitivity estimates of approximately 0.70 for the Awaji criteria and 0.58 for the rEEC criteria, with a clearer separation between the two approaches [38]. In contrast, the present study estimated a sensitivity of 0.87 for both criteria, with no statistically significant difference between them. The findings are therefore not quantitatively concordant, although both studies indicate that the Awaji criteria are not less sensitive than the rEEC criteria. Differences in the absolute estimates may reflect variation in study populations, eligibility criteria, disease spectrum, and reference-standard classification.

This IPD meta-analysis has several strengths, including comprehensive searches, verification of shared data against published results, and author contact to resolve discrepancies and missing information. All five studies that met the review-level eligibility criteria provided IPD. Therefore, inclusion in the meta-analysis was determined by study eligibility rather than IPD availability. Access to IPD enabled outcome harmonisation, covariate adjustment, and subgroup analyses to test robustness. The consistently higher sensitivity of GCC, with acceptably lower specificity and low-to-very low certainty of evidence, supported by robust analyses, underpins its diagnostic utility in ALS. Inclusion of large cohorts from diverse regions supports applicability in specialist neurologist settings.

An important limitation of the present meta-analysis is the absence of an independent, pathognomonic reference standard for ALS, resulting in heterogeneity in the reference standards used across the included cohorts. Although all studies sought to establish a final diagnosis of ALS or non-ALS through longitudinal clinical assessment, the approaches varied across cohorts and included clinical progression during follow-up, diagnosis at the last follow-up, attending-physician judgement, and classification as non-ALS following identification of an alternative diagnosis or the absence of progression. This reflects real-world ALS diagnostic practice but also introduces potential diagnostic review and incorporation bias because expert clinical adjudication relies on similar clinical and neurophysiological features evaluated by the index criteria. Consequently, the apparent diagnostic accuracy of all three criteria may have been overestimated. Statistical modelling can improve the precision and comparability of diagnostic-performance estimates but cannot eliminate uncertainty arising from an imperfect clinical reference standard. The pooled estimates should therefore be interpreted as measures of comparative performance against real-world expert clinical adjudication rather than as absolute measures of diagnostic truth. This limitation may be particularly relevant to specificity because false-positive classification is influenced by the composition of the mimic population, the duration of follow-up, the investigations undertaken, and the clinical threshold used to distinguish ALS from non-ALS disorders. Accordingly, pooled specificity estimates should be regarded as setting-dependent rather than fixed properties of the criteria. A further limitation is that the number of studies evaluating the diagnostic accuracy of GCC is small given that the GCC were proposed relatively recently [13], potentially representing a limitation. Of thirteen studies identified as potentially eligible at initial screening, only five met the inclusion criteria (Supplementary Fig. 1). Additionally, the restriction to English-language publications may have introduced a potential risk of language bias. In addition, missing data were not consistently quantified by individual variable in the included studies, precluding formal assessment of the extent or effect of missingness on the pooled estimates. Of relevance, several review authors were involved in developing the Gold Coast criteria, contributing to included cohorts, or developing national MND guidance. Although these relationships were disclosed and the review followed prespecified analytical methods, this intellectual overlap represents a potential source of allegiance bias and may have influenced the interpretation or framing of the findings.

Specificity estimates should also be interpreted cautiously, as the size and characterisation of non-ALS comparator cohorts varied substantially across studies. These factors likely contributed to imprecision and between-study heterogeneity in specificity estimates. The IPD provided for Pugdahl and colleagues [19] included participants aged > 80 years and/or with symptom duration > 36 months who were outside the eligibility criteria applied in the original publication but met the prespecified review-level eligibility criteria. Although their inclusion could have affected comparability with the published Pugdahl cohort, restricting the analysis to the originally published eligibility criteria did not materially alter the pooled estimates. Although involvement of some authors in the development of the Gold Coast criteria and included cohorts may represent a potential source of bias, rigorous prespecified methods and transparent reporting helped minimise the potential influence of such bias on the interpretation of findings.

### Clinical implications

A key implication of this study is that GCC improve diagnostic sensitivity in suspected ALS and may facilitate earlier access to ALS-specific care when applied in specialist neuromuscular settings. Earlier diagnosis may allow earlier initiation of riluzole, timely multidisciplinary care, respiratory and nutritional surveillance, genetic counselling where appropriate, and access to therapeutic trials. These benefits should not be interpreted as equivalent to the treatment effect observed in riluzole trials, as the relevant comparison is earlier versus later diagnosis rather than treatment versus no treatment. The lower and more variable specificity estimates also highlight the potential harms of false-positive ALS classification, that may delay diagnosis and treatment of mimic disorders. Application of the Gold Coast criteria should include clinically appropriate investigations to exclude alternative diagnoses, as explicitly required by the criteria, together with longitudinal reassessment when diagnostic uncertainty persists.

Identification of upper motor neuron involvement remains a major diagnostic challenge in ALS [39]. Neurophysiological approaches, including threshold tracking transcranial magnetic stimulation, may provide objective markers of cortical dysfunction [3, 40–43], although wider clinical implementation remains limited and requires further validation across centres. Similarly, neurofilament light chain (NfL) is a robust marker of neuroaxonal injury and disease activity in ALS, but its diagnostic utility is limited by lack of disease specificity and should not be considered in isolation [44, 45]. The lower and heterogeneous specificity of the GCC may potentially be improved by integrating clinical criteria with adjunctive biomarkers. Emerging studies provide preliminary support for such a multimodal approach [46]. In a prospective cohort study, Calma and colleagues reported that combining the GCC with elevated serum NfL or cortical dysfunction increased specificity from 0.89 to 0.98 while maintaining high sensitivity at 0.87[46]. Serum NfL integrated with the GCC has also been associated with substantially greater odds of identifying patients who subsequently progressed to definite ALS [47], while combining serum NfL with cardiac troponin T improved discrimination between ALS and disease controls [48]. These findings are promising but require cautious interpretation because of differences in study populations, comparator groups, biomarker thresholds and reference standards, as well as the limited external validation of some approaches. Larger prospective multicentre studies are therefore required to determine whether multimodal diagnostic algorithms provide reproducible improvements in specificity over GCC alone and how they should be implemented in routine clinical practice. In conclusion, the present IPD meta-analysis supports GCC as sensitive diagnostic criteria for ALS. Their application should not curtail investigation for mimic disorders, particularly in atypical or lower motor neuron-predominant presentations. When used within an expert diagnostic framework, GCC may facilitate earlier diagnosis, earlier consideration of riluzole, timely multidisciplinary management, respiratory and nutritional surveillance, genetic counselling where appropriate, and recruitment into therapeutic trials. Further prospective studies are required to define their specificity in well-characterised mimic populations and to determine whether adjunctive biomarkers can improve diagnostic confidence across diverse clinical settings.

## Supplementary Information

Below is the link to the electronic supplementary material.

Supplementary file1 (DOCX 762 KB)

## Data Availability

This study did not generate new datasets. The individual participant data analysed in this study were provided by the corresponding authors of the included studies and are not publicly available. Requests for access should be directed to the investigators of the original studies.
