# Supplementary material for: Gold Coast criteria for ALS diagnosis: individual participant data meta-analysis
Source: J Neurol. 2026 Aug 19;273(9):532. doi: 10.1007/s00415-026-14046-y (PMC13489995; doi:10.1007/s00415-026-14046-y)
Supplement: Supplementary file 1 [file 415_2026_14046_MOESM1_ESM.docx]

**Gold Coast Criteria for Amyotrophic Lateral Sclerosis Diagnosis:** **A systematic review and individual participant data meta-analysis**

Supplementary Figure 1: PRISMA flow chart

Supplementary Figure 2: Subgroup Forest plot for borderline ALS- Gold Coast vs. Awaji criteria

Supplementary Figure 3: Subgroup Forest plot for borderline ALS- Gold Coast vs. revised EL Escorial criteria

Supplementary Figure 4: QUADAS summary

Supplementary Table 1: Primary lateral sclerosis cases in database

Supplementary Table 2: Excluded studies

Supplementary Table 3: Participant characteristics

Supplementary Table 4: Sensitivity analysis

Supplementary Table 5: Should Gold Coast criteria vs. Awaji criteria be used to diagnose Amyotrophic lateral sclerosis in patients presenting with signs and symptoms of amyotrophic lateral sclerosis?

Supplementary Table 6: Should Gold Coast criteria vs. Awaji criteria be used to diagnose Amyotrophic lateral sclerosis in patients presenting with signs and symptoms of amyotrophic lateral sclerosis?

Supplementary 1: Search Strategy

Supplementary 2: Setting decision thresholds using downstream health outcomes for summary of findings table

Supplementary 3: QUADAS-2 review-specific guidance

Supplementary 4: QUADAS-2 and QUADAS-C Judgement tables

Supplementary 5: Author responses to QUADAS questions

**Supplementary Figure 1: PRISMA flow chart**

**Screening**

**Included**

**IPD acquisition**

Reports assessed in full text for eligibility (n=13)

Records Identified from databases

(n=114)

Medline (n=30)

Scopus (n=48)

Embase (n=36)

Records removed before screening:

Duplicate records removed (n=77)

**Identification**

Records screened (title and abstract) (n=37)

Records excluded (n=24)

Studies included in review **(n=5)**

Reports excluded:

Wrong study design (n=3)

Wrong comparator (n=1)

Wrong patient population (n=1)

Wrong reference standard/no non-ALS population (n=3)

Studies eligible for individual participant data (IPD); n=5

IPD not obtained (n=0)

**Supplementary Figure 2: Subgroup Forest plot for borderline ALS- Gold Coast vs. Awaji criteria**

**Supplementary Figure 3: Subgroup Forest plot for borderline ALS- Gold Coast vs. revised EL Escorial criteria**

**Supplementary Figure 4: QUADAS summary**

| **Study** | **Risk of bias**  **(QUADAS-2)** | | | | | |  | **Applicability concerns**  **(QUADAS-2)** | | | | |  | **Risk of bias**  **(QUADAS-C)** | | | |
| --- | --- | --- | --- | --- | --- | --- | --- | --- | --- | --- | --- | --- | --- | --- | --- | --- | --- |
|  | **P** | **I** | | | **R** | **FT** |  | **P** | **I** | | | **R** |  | **P** | **I** | **R** | **FT** |
|  |  | **GC** | **AW** | **rEEC** |  |  |  |  | **GC** | **AW** | **rEEC** |  |  |  |  |  |  |
| **Ferullo 2024** | ✓ | ✗ | ✗ | ✗ | ✓ | ✓ |  | ✓ | ✓ | ✓ | ✓ | ✓ |  | ✓ | ✗ | ✓ | ✓ |
| **Hannaford 2021** | ✓ | ✓ | ✓ | ✓ | ✓ | ✓ |  | ✓ | ✓ | ✓ | ✓ | ✓ |  | ✓ | ✓ | ✓ | ✓ |
| **Otani 2024** | ✓ | ? | ? | ? | ✓ | ✓ |  | ✓ | ✓ | ✓ | ✓ | ✓ |  | ✓ | ? | ✓ | ✓ |
| **Pugdahl 2021** | ✓ | ✓ | ✓ | ✓ | ✓ | ? |  | ✓ | ✓ | ✓ | ✓ | ✓ |  | ✓ | ✓ | ✓ | ? |
| **Shen 2021** | ? | ✓ | ✓ | ✓ | ✓ | ? |  | ✓ | ✓ | ✓ | ✓ | ✓ |  | ? | ✓ | ✓ | ? |

P = patient selection; I = index test; R = reference standard; FT = flow and timing.
✓ indicates low risk; ✗ indicates high risk; ? indicates unclear risk.

GC= Gold Coast Criteria; AW = Awaji Criteria; rEEC = Revised El Escorial Criteria

**Supplementary Table 1: Primary lateral sclerosis cases in database**

| Study | PLS cases | GCC positive | Awaji positive | rEEC positive | Classified as ALS according to reference standard | Handling in primary analysis |
| --- | --- | --- | --- | --- | --- | --- |
| Hannaford | 19 | 0 | 0 | 0 | 19 | Non-ALS |
| Shen | 0 | 0 | 0 | 0 | 0 | Non-ALS |
| Otani | 5 | 0 | 5 | 5 | 0 | Non-ALS |
| Pugdahl | 1 | 0 | 1 | 1 | 0 | Non-ALS |
| Ferullo | 0 | 0 | 0 | 0 | 0 | Non-ALS |

**Supplementary Table 2: Excluded studies**

| Study ID | Reason for exclusion | Inclusion in published aggregate data meta-analysis |
| --- | --- | --- |
| Jewett 2022[1] | Only ALS participants were recruited. No reference standard. Retrospective design.  IPD was provided | Included in von Quednow et al. (2025) |
| Ohnari 2025[2] | Only ALS participants were recruited. No reference standard. Retrospective design.  IPD not provided | Not included |
| De Jongh 2022[3] | Only ALS participants were recruited. No reference standard. Prospective design.  IPD was provided | Included in von Quednow et al. (2025) |
| Desmaison 2024[4] | Wrong index test: Investigated whether incorporating transcranial magnetic stimulation improves diagnostic accuracy when added to the Awaji and Gold Coast criteria. Prospective | Included in von Quednow et al. (2025) |
| Laucius 2025[5] | Wrong index test: Examined ultrasonographic changes in the vagus nerve as a supplementary diagnostic approach for ALS—prospective design. | Not included |
| Vucic 2021[6] | Wrong study design: Literature review | Not included |
| Ortiz-Corredor 2025[7] | Wrong study design: Investigated the prognostic value of the Amyotrophic Lateral Sclerosis Functional Rating Scale-Revised (ALSFRS-R) in predicting mortality and characterizing disease progression patterns in ALS patients in Colombia—retrospective design. | Not included |
| Stikvoort Garcia 2023[8] | Wrong index test: Investigated the diagnostic accuracy of a novel neurophysiological protocol in patients with suspected ALS—prospective design. | Included in von Quednow et al. (2025) |

\# ALS – Amyotrophic lateral sclerosis, ALSFRS-R – Amyotrophic lateral sclerosis Functional rating scale. Where wrong index test or study design were identified on screening the manuscript, Individual patients data (IPD) was not sought.

**Supplementary Table 3: Participants' characteristics**

| **Characteristic** | **Overall**  N = 3,007^1^ | **Non-ALS**  N = 564^1^ | **ALS**  N = 2,443^1^ |
| --- | --- | --- | --- |
| **Age** |  |  |  |
| Median (Q1-Q3) | 60.0 (51.0-69.0) | 63.0 (51.5-71.9) | 60.0 (51.0-69.0) |
| Mean (SD) | 59.6 (13.1) | 60.7 (14.8) | 59.3 (12.6) |
| % Missing% | 0.2% | 0.0% | 0.3% |
| **Sex** |  |  |  |
| Female | 1,323 (44%) | 218 (39%) | 1,105 (45%) |
| Male | 1,684 (56%) | 346 (61%) | 1,338 (55%) |
| **Site of disease onset** |  |  |  |
| Bulbar | 725 (25%) | 48 (12%) | 677 (28%) |
| Spinal | 2,123 (75%) | 360 (88%) | 1,763 (72%) |
| **Duration of disease (months)** |  |  |  |
| Median (Q1-Q3) | 12.0 (8.0-23.0) | 23.0 (9.0-48.0) | 12.0 (8.0-20.0) |
| Mean (SD) | 20.0 (26.2) | 39.0 (50.9) | 16.8 (17.3) |
| % Missing% | 6.1% | 28.9% | 0.8% |
| **ALSFRS** |  |  |  |
| Median (Q1-Q3) | 40.0 (35.0-44.0) | 43.0 (37.5-46.0) | 40.0 (35.0-44.0) |
| Mean (SD) | 38.5 (6.9) | 41.1 (6.6) | 38.5 (6.9) |
| % Missing% | 28.8% | 87.2% | 15.3% |
| ^1^n (%) | | | |

\# ALS – Amyotrophic lateral sclerosis, ALSFRS-R – Amyotrophic lateral sclerosis Functional rating scale

**Supplementary Table 4: Sensitivity analysis**

| Criteria | Number of studies | Number of participants | Sensitivity | | | Specificity | | | Area under curve |
| --- | --- | --- | --- | --- | --- | --- | --- | --- | --- |
|  |  |  | **Pooled estimate** | **ͳ^2^** | **Prediction interval** | **Pooled estimate** | **ͳ^2^** | **Prediction interval** |  |
| **One stage model: Leave one out analysis-excluding Ferullo 2024** | | | | | | | | |  |
| Gold Coast | 4 | 2772 | 0.96 (95% CI 0.93 to 0.97) | 0.63 | 0.85 to 0.99 | 0.82 (95% CI 0.75 to 0.88) | 1.30 | 0.02 to 0.88 |  |
| Awaji |  |  | 0.86 (95% CI 0.80 to 0.90) | 0.38 | 0.74 to 0.94 | 0.79 (95% CI 0.70 to 0.85) | 1.00 | 0.04 to 0.85 |  |
| Revised El Escorial |  |  | 0.85 (95% CI 0.79 to 0.90) | 0.36 | 0.74 to 0.93 | 0.79 (95% CI 0.70 to 0.86) | 1.01 | 0.04 to 0.86 |  |
| **One stage model: Leave one out analysis-excluding Shen 2021** | | | | | | | | |  |
| Gold Coast | 4 | 1822 | 0.96 (95% CI 0.91 to 0.98) | 0.67 | 0.81 to 0.99 | 0.71 (95% CI 0.51 to 0.86) | 1.94 | 0.01 to 0.97 |  |
| Awaji |  |  | 0.90 (95% CI 0.78 to 0.95) | 0.46 | 0.68 to 0.95 | 0.75 (95% CI 0.56 to 0.88) | 0.94 | 0.04 to 0.88 |  |
| Revised El Escorial |  |  | 0.88 (95% CI 0.77 to 0.94) | 0.51 | 0.62 to 0.94 | 0.76 (95% CI 0.58 to 0.87) | 0.92 | 0.04 to 0.87 |  |
| **One stage model: Restricting Pugdal 2021 data as per the eligibility criteria reported in the publication** | | | | | | | | |  |
| Gold Coast | 5 | 2927 | 0.96 (95% CI 0.93 to 0.98) | 0.56 | 0.87 to 0.99 | 0.66 (95% CI 0.51 to 0.78) | 1.8 | 0.02 to 0.97 |  |
| Awaji |  |  | 0.88 (95% CI 0.80 to 0.93) | 0.38 | 0.73 to 0.93 | 0.70 (95% CI 0.56 to 0.81) | 0.93 | 0.07 to 0.81 |  |
| Revised El Escorial |  |  | 0.86 (95% CI 0.79 to 0.92) | 0.43 | 0.68 to 0.93 | 0.70 (95% CI 0.58 to 0.80) | 0.92 | 0.07 to 0.80 |  |

**Supplementary Table 5: Should Gold Coast criteria vs. Awaji criteria be used to diagnose Amyotrophic lateral sclerosis in patients presenting with signs and symptoms of amyotrophic lateral sclerosis?**

| Gold Coast criteria | | Awaji criteria | |
| --- | --- | --- | --- |
| Sensitivity | 0.96 (95% CI: 0.93 to 0.98) | Sensitivity | 0.87 (95% CI: 0.78 to 0.93) |
| Specificity | 0.68 (95% CI: 0.53 to 0.81) | Specificity | 0.72 (95% CI: 0.57 to 0.83) |

| Prevalences | 25% | 50% | 75% |
| --- | --- | --- | --- |

| Pre-test probability | Outcome | № of studies (№ of patients) | Study design | Factors that may decrease certainty of evidence | | | | | Effect per 1,000 patients tested | | Test accuracy CoE |
| --- | --- | --- | --- | --- | --- | --- | --- | --- | --- | --- | --- |
|  |  |  |  | Risk of bias | Indirectness | Inconsistency | Imprecision | Publication bias | Gold Coast criteria | Awaji criteria |  |
| **25%** | **True positives** (patients with Amyotrophic lateral sclerosis) | 5 studies 3007 patients | cross-sectional (cohort type accuracy study) | serious^a^ | not serious | not serious | serious^b^ | none | 240 (233 to 245) | 218 (195 to 233) | ⨁⨁◯◯ Low^a,b^ |
|  |  |  |  |  |  |  |  |  | **22 more TP in Gold Coast criteria** | |  |
|  | **False negatives** (patients incorrectly classified as not having Amyotrophic lateral sclerosis) |  |  |  |  |  |  |  | 10 (5 to 17) | 32 (17 to 55) |  |
|  |  |  |  |  |  |  |  |  | **22 fewer FN in Gold Coast criteria** | |  |
|  | **True negatives** (patients without Amyotrophic lateral sclerosis) | 5 studies 3007 patients | cross-sectional (cohort type accuracy study) | serious^a^ | not serious | not serious^c^ | very serious^b^ | none | 510 (398 to 608) | 540 (428 to 622) | ⨁◯◯◯ Very low^a,b,c^ |
|  |  |  |  |  |  |  |  |  | **30 fewer TN in Gold Coast criteria** | |  |
|  | **False positives** (patients incorrectly classified as having Amyotrophic lateral sclerosis) |  |  |  |  |  |  |  | 240 (142 to 352) | 210 (128 to 322) |  |
|  |  |  |  |  |  |  |  |  | **30 more FP in Gold Coast criteria** | |  |
| **50%** | **True positives** (patients with Amyotrophic lateral sclerosis) | 5 studies 3007 patients | cross-sectional (cohort type accuracy study) | serious^a^ | not serious | not serious | not serious^d^ | none | 480 (465 to 490) | 435 (390 to 465) | ⨁⨁⨁◯ Moderate^a,d^ |
|  |  |  |  |  |  |  |  |  | **45 more TP in Gold Coast criteria** | |  |
|  | **False negatives** (patients incorrectly classified as not having Amyotrophic lateral sclerosis) |  |  |  |  |  |  |  | 20 (10 to 35) | 65 (35 to 110) |  |
|  |  |  |  |  |  |  |  |  | **45 fewer FN in Gold Coast criteria** | |  |
|  | **True negatives** (patients without Amyotrophic lateral sclerosis) | 5 studies 3007 patients | cross-sectional (cohort type accuracy study) | serious^a^ | not serious | not serious^c^ | very serious^d^ | none | 340 (265 to 405) | 360 (285 to 415) | ⨁◯◯◯ Very low^a,c,d^ |
|  |  |  |  |  |  |  |  |  | **20 fewer TN in Gold Coast criteria** | |  |
|  | **False positives** (patients incorrectly classified as having Amyotrophic lateral sclerosis) |  |  |  |  |  |  |  | 160 (95 to 235) | 140 (85 to 215) |  |
|  |  |  |  |  |  |  |  |  | **20 more FP in the Gold Coast criteria** | |  |
| **75%** | **True positives** (patients with Amyotrophic lateral sclerosis) | 5 studies 3007 patients | cross-sectional (cohort type accuracy study) | serious^a^ | not serious | not serious | not serious | none | 720 (698 to 735) | 653 (585 to 698) | ⨁⨁⨁◯ Moderate^a,e^ |
|  |  |  |  |  |  |  |  |  | **67 more TP in Gold Coast criteria** | |  |
|  | **False negatives** (patients incorrectly classified as not having Amyotrophic lateral sclerosis) |  |  |  |  |  |  |  | 30 (15 to 52) | 97 (52 to 165) |  |
|  |  |  |  |  |  |  |  |  | **67 fewer FN in Gold Coast criteria** | |  |
|  | **True negatives** (patients without Amyotrophic lateral sclerosis) | 5 studies 3007 patients | cross-sectional (cohort type accuracy study) | serious^a^ | not serious | not serious^c^ | very serious^e^ | none | 170 (133 to 203) | 180 (143 to 208) | ⨁◯◯◯ Very low^a,c,e^ |
|  |  |  |  |  |  |  |  |  | **10 fewer TN in Gold Coast criteria** | |  |
|  | **False positives** (patients incorrectly classified as having Amyotrophic lateral sclerosis) |  |  |  |  |  |  |  | 80 (47 to 117) | 70 (42 to 107) |  |
|  |  |  |  |  |  |  |  |  | **10 more FP in the Gold Coast criteria** | |  |

a. Only one study in the pooled analysis was at low risk, three studies at unclear risk and the remaining one study at high risk.
b. For 25% pre-test probability

For TP with 95% CI 15 to 30; the downstream outcome number of lives saved ranges from 10 to 20. Considering the small benefit in mortality of 13 per 1000; the plausible range of TP extends from no benefit to small benefit; therefore, downgraded one level for imprecision.

For FP with 95% CI 0 to 130; the downstream outcome adverse event ranges from 0 to 97. Considering the small harm in adverse events of 30 per 1000 and large harm of 64 per 1000, the plausible range of FP ranges from no harm to large harm; therefore, downgraded two levels for imprecision.

c. Confidence intervals of individual studies do not overlap and wide prediction intervals. Not downgraded as downgraded for inconsistency and additional downgrading is not possible.
d. For 50% pre-test probability

For TP with 95% CI 30 to 55; the downstream outcome number of lives saved ranges from 20 to 36. Considering the small benefit in mortality of 13 per 1000 and the moderate benefit of 32 per 1000, the plausible range of TP extends from small to moderate benefit; therefore, not downgraded for imprecision, given at least a small mortality benefit.

For FP with 95% CI 0 to 95; the downstream outcome adverse event ranges from 0 to 73. Considering the small harm in adverse events of 30 per 1000 and large harm of 64 per 1000, the plausible range of FP ranges from no harm to large harm; therefore, downgraded two levels for imprecision.

e. For 75% pre-test probability

For TP with 95% CI 45 to 82; the downstream outcome number of lives saved ranges from 30 to 56. Considering the small mortality benefit of 13 per 1000, the plausible range of TP extends from small to moderate benefit; therefore, not downgraded for imprecision, given at least a small mortality benefit.

For FP with 95% CI 0 to 50; the downstream outcome adverse event ranges from 0 to 37. Considering the small harm in adverse events of 30 per 1000, the plausible FP range is from no harm to moderate; therefore, downgraded two levels for imprecision.

**Supplementary Table 6: Should Gold Coast criteria vs. revised El Escorial criteria be used to diagnose Amyotrophic lateral sclerosis in patients presenting with signs and symptoms of amyotrophic lateral sclerosis?**

| Gold Coast criteria | | Revised El Escorial criteria | |
| --- | --- | --- | --- |
| Sensitivity | 0.96 (95% CI: 0.93 to 0.98) | Sensitivity | 0.87 (95% CI: 0.78 to 0.92) |
| Specificity | 0.68 (95% CI: 0.53 to 0.81) | Specificity | 0.73 (95% CI: 0.59 to 0.83) |

| Prevalences | 25% | 50% | 75% |
| --- | --- | --- | --- |

| Pre-test probability | Outcome | № of studies (№ of patients) | Study design | Factors that may decrease certainty of evidence | | | | | Effect per 1,000 patients tested | | Test accuracy CoE |
| --- | --- | --- | --- | --- | --- | --- | --- | --- | --- | --- | --- |
|  |  |  |  | Risk of bias | Indirectness | Inconsistency | Imprecision | Publication bias | Gold Coast criteria | Revised El Escorial criteria |  |
| **25%** | **True positives** (patients with Amyotrophic lateral sclerosis) | 5 studies 3007 patients | cross-sectional (cohort type accuracy study) | serious^a^ | not serious | not serious | serious^b^ | none | 240 (233 to 245) | 218 (195 to 230) | ⨁⨁◯◯ Low^a,b^ |
|  |  |  |  |  |  |  |  |  | **22 more TP in Gold Coast criteria** | |  |
|  | **False negatives** (patients incorrectly classified as not having Amyotrophic lateral sclerosis) |  |  |  |  |  |  |  | 10 (5 to 17) | 32 (20 to 55) |  |
|  |  |  |  |  |  |  |  |  | **22 fewer FN in Gold Coast criteria** | |  |
|  | **True negatives** (patients without Amyotrophic lateral sclerosis) | 5 studies 3007 patients | cross-sectional (cohort type accuracy study) | serious^a^ | not serious | not serious^c^ | very serious^b^ | none | 510 (398 to 608) | 548 (443 to 622) | ⨁◯◯◯ Very low^a,b,c^ |
|  |  |  |  |  |  |  |  |  | **38 fewer TN in Gold Coast criteria** | |  |
|  | **False positives** (patients incorrectly classified as having Amyotrophic lateral sclerosis) |  |  |  |  |  |  |  | 240 (142 to 352) | 202 (128 to 307) |  |
|  |  |  |  |  |  |  |  |  | **38 more FP in Gold Coast criteria** | |  |
| **50%** | **True positives** (patients with Amyotrophic lateral sclerosis) | 5 studies 3007 patients | cross-sectional (cohort type accuracy study) | serious^a^ | not serious | not serious | not serious^d^ | none | 480 (465 to 490) | 435 (390 to 460) | ⨁⨁⨁◯ Moderate^a,d^ |
|  |  |  |  |  |  |  |  |  | **45 more TP in Gold Coast criteria** | |  |
|  | **False negatives** (patients incorrectly classified as not having Amyotrophic lateral sclerosis) |  |  |  |  |  |  |  | 20 (10 to 35) | 65 (40 to 110) |  |
|  |  |  |  |  |  |  |  |  | **45 fewer FN in Gold Coast criteria** | |  |
|  | **True negatives** (patients without Amyotrophic lateral sclerosis) | 5 studies 3007 patients | cross-sectional (cohort type accuracy study) | serious^a^ | not serious | not serious^c^ | very serious^d^ | none | 340 (265 to 405) | 365 (295 to 415) | ⨁◯◯◯ Very low^a,c,d^ |
|  |  |  |  |  |  |  |  |  | **25 fewer TN in Gold Coast criteria** | |  |
|  | **False positives** (patients incorrectly classified as having Amyotrophic lateral sclerosis) |  |  |  |  |  |  |  | 160 (95 to 235) | 135 (85 to 205) |  |
|  |  |  |  |  |  |  |  |  | **25 more FP in Gold Coast criteria** | |  |
| **75%** | **True positives** (patients with Amyotrophic lateral sclerosis) | 5 studies 3007 patients | cross-sectional (cohort type accuracy study) | serious^a^ | not serious | not serious | not serious^e^ | none | 720 (698 to 735) | 653 (585 to 690) | ⨁⨁⨁◯ Moderate^a,e^ |
|  |  |  |  |  |  |  |  |  | **67 more TP in Gold Coast criteria** | |  |
|  | **False negatives** (patients incorrectly classified as not having Amyotrophic lateral sclerosis) |  |  |  |  |  |  |  | 30 (15 to 52) | 97 (60 to 165) |  |
|  |  |  |  |  |  |  |  |  | **67 fewer FN in Gold Coast criteria** | |  |
|  | **True negatives** (patients without Amyotrophic lateral sclerosis) | 5 studies 3007 patients | cross-sectional (cohort type accuracy study) | serious^a^ | not serious | not serious^c^ | very serious^e^ | none | 170 (133 to 203) | 183 (148 to 208) | ⨁◯◯◯ Very low^a,c,e^ |
|  |  |  |  |  |  |  |  |  | **13 fewer TN in Gold Coast criteria** | |  |
|  | **False positives** (patients incorrectly classified as having Amyotrophic lateral sclerosis) |  |  |  |  |  |  |  | 80 (47 to 117) | 67 (42 to 102) |  |
|  |  |  |  |  |  |  |  |  | **13 more FP in Gold Coast criteria** | |  |

a. Only one study in the pooled analysis was at low risk, one study at unclear risk and the remaining three studies at high risk.
b. For 25% pre-test probability

For TP with 95% CI 15 to 27; the downstream outcome number of lives saved ranges from 10 to 18. Considering the small benefit in mortality of 13 per 1000; the plausible range of TP extends from no benefit to small benefit; therefore, downgraded one level for imprecision.

For FP with 95% CI 0 to 150; the downstream outcome adverse event ranges from 0 to 112. Considering the small harm in adverse events of 30 per 1000 and large harm of 64 per 1000; the plausible range of FP ranges from no harm to large harm; therefore, downgraded two levels for imprecision.

c. Confidence intervals of individual studies do not overlap and wide prediction intervals. Not downgraded as downgraded for inconsistency and additional downgrading not possible.
d. For 50% pre-test probability

For TP with 95% CI 30 to 55; the downstream outcome number of lives saved ranges from 20 to 37. Considering the small benefit in mortality of 13 per 1000 and the moderate benefit of 32 per 1000, the plausible range of TP extends from small to moderate benefit; therefore, not downgraded for imprecision, given at least a small mortality benefit.

For FP with 95% CI 0 to 100; the downstream outcome adverse event ranges from 0 to 75. Considering the small harm in adverse events of 30 per 1000 and moderate harm of 74 per 1000, the plausible range of FP ranges from no harm to moderate harm; therefore, downgraded two levels for imprecision.

e. For 75% pre-test probability

For TP with 95% CI 45 to 82; the downstream outcome number of lives saved ranges from 30 to 56. Considering the small benefit in mortality of 13 per 1000 and the moderate benefit of 32 per 1000, the plausible range of TP extends from small to moderate benefit; therefore, not downgraded for imprecision, given at least a small mortality benefit.

For FP with 95% CI 0 to 50; the downstream outcome adverse event ranges from 0 to 37. Considering the small harm in adverse events of 30 per 1000, the plausible FP range is from no harm to moderate harm; therefore, downgraded one level for imprecision.

**Supplementary 1: Search Strategy**

**MEDLINE**

| **Step** | **Search terms** |
| --- | --- |
| 1 | "Gold Coast criteria" |
| 2 | "Awaji criteria" |
| 3 | "El Escorial criteria" |
| 4 | "revised El Escorial" OR "Airlie House criteria" |
| 5 | #2 OR #3 OR #4 |
| 6 | #1 AND #5 |
| 7 | "Amyotrophic Lateral Sclerosis"[MeSH] OR "Motor Neuron Disease"[MeSH] |
| 8 | "amyotrophic lateral sclerosis" OR "motor neuron disease" |
| 9 | #7 OR #8 |
| 10 | #6 AND #9 |
| 11 | Filters: Humans; Publication dates 1 January 2020 to 1 October 2025; English language |

**SCOPUS**

| **Step** | **Search terms** |
| --- | --- |
| 1 | TITLE-ABS-KEY ("Gold Coast criteria") |
| 2 | TITLE-ABS-KEY ("Awaji criteria") |
| 3 | TITLE-ABS-KEY ("El Escorial criteria") |
| 4 | TITLE-ABS-KEY ("revised El Escorial" OR "Airlie House criteria") |
| 5 | #2 OR #3 OR #4 |
| 6 | #1 AND #5 |
| 7 | TITLE-ABS-KEY ("amyotrophic lateral sclerosis" OR ALS OR "motor neuron disease") |
| 8 | #6 AND #7 |
| 9 | Filters: Humans; Publication years 2020–2025; English language |

**EMBASE via OVID**

| **Step** | **Search terms** |
| --- | --- |
| 1 | 'Gold Coast criteria'.ti,ab,kw. |
| 2 | 'awaji criteria'.ti,ab,kw. |
| 3 | 'El Escorial criteria'.ti,ab,kw. |
| 4 | ('revised el escorial' OR 'airlie house criteria').ti,ab,kw. |
| 5 | #2 OR #3 OR #4 |
| 6 | #1 AND #5 |
| 7 | 'amyotrophic lateral sclerosis'/exp OR 'motor neuron disease'/exp |
| 8 | ('amyotrophic lateral sclerosis' OR ALS OR 'motor neuron disease').ti,ab,kw. |
| 9 | #7 OR #8 |
| 10 | #6 AND #9 |
| 11 | Limits: Humans; Publication years 2020–2025; English language |

**Supplementary 2: Setting decision thresholds using downstream health outcomes for summary of findings table**

We used decision thresholds calculated for downstream health outcomes mortality and adverse events for the Australian Motor Neurone Disease Guideline.[9]

**Decision thresholds to inform the Australian MND Guideline**

| **Outcome** | **Trivial to Small** | **Small to Moderate** | **Moderate to Large** |
| --- | --- | --- | --- |
| **Adverse events** | 30 per 1000 | 74 per 1000 | 144 per 1000 |
| **Mortality** | 13 per 1000 | 32 per 1000 | 64 per 1000 |

We modelled how many lives are saved and how many more adverse events are expected when using the Gold Coast criteria compared with the Awaji/Revised El Escorial criteria for each pre-test probability, using the evidence profile generated for the Australian motor neuron disease guideline on the effectiveness of riluzole. These analyses were intended to provide illustrative estimates of possible clinical consequences rather than direct estimates of mortality benefit attributable to the Gold Coast criteria.

For true-positive ALS cases, earlier diagnosis may increase the opportunity for earlier initiation of riluzole, multidisciplinary care, respiratory and nutritional surveillance, genetic counselling where appropriate, and access to therapeutic trials. For false-negative cases, diagnosis may be delayed, and these patients may not receive these interventions as early. Diagnostic delays in ALS have been reported to range from 9.1 to 27 months.[10] In the model, true-positive cases were assumed to have the opportunity to benefit from earlier initiation of riluzole. In contrast, false-negative cases were assumed not to receive this early treatment benefit within the modelled time horizon.

ALS-specific mortality was not applied to true-negative or false-positive groups because these individuals did not have ALS according to the reference standard. However, false-positive cases may be exposed to unnecessary ALS-specific treatment, including riluzole-related adverse events. More importantly, false-positive ALS classification may also cause psychological harm, inappropriate counselling, therapeutic trial misclassification, and delayed recognition or treatment of alternative diagnoses, including potentially treatable mimic disorders. These harms are not fully captured by riluzole adverse-event rates alone.

These estimates should therefore be interpreted cautiously. The magnitude of any clinical benefit from earlier diagnosis depends on the time gained, treatment uptake, patient phenotype, access to specialist multidisciplinary care, and the accuracy with which ALS mimic disorders are excluded.

**For Gold Coast vs. Awaji criteria**

**For 25% pre-test probability:**

Number of lives saved = relative risk*absolute increase in true positive

= 0.68* 22 = 15 (95% CI 10 to 20)

Number of adverse events = baseline risk in riluzole group * absolute increase in false positive

= 0.746 *30=22 (95% CI 0 to 97)

**For 50% pre-test probability:**

Number of lives saved = relative risk*absolute increase in true positive

=0.68*45 = 30 (95% CI 20 to 36)

Number of adverse events = baseline risk in riluzole group * absolute increase in false positive

= 0.746 * 20 = 15 (95% CI 0 to 73)

**For 75% pre-test probability:**

Number of lives saved = relative risk*absolute increase in true positive

=0.68*67 = 45 (95% CI 30 to 56)

Number of adverse events = baseline risk in riluzole group * absolute increase in false positive

= 0.746 * 10 = 20 (95% CI 0 to 37)

**For Gold Coast vs. revised El Escorial criteria**

**For 25% pre-test probability:**

Number of lives saved = relative risk*absolute increase in true positive

= 0.68* 22 = 15 (95% CI 10 to 18)

Number of adverse events = baseline risk in riluzole group * absolute increase in false positive

= 0.746 *38=28 (95% CI 0 to 112)

**For 50% pre-test probability:**

Number of lives saved = relative risk*absolute increase in true positive

=0.68*45 = 30 (95% CI 20 to 37)

Number of adverse events = baseline risk in riluzole group * absolute increase in false positive

= 0.746 * 25 = 15 (95% CI 0 to 75)

**For 75% pre-test probability:**

Number of lives saved = relative risk*absolute increase in true positives

=0.68*67 = 45 (95% CI 30 to 56)

Number of adverse events = baseline risk in riluzole group * absolute increase in false positives

= 0.746 * 13 = 20 (95% CI 0 to 55)

**Summary of Findings: Riluzole 100mg versus control**

| **Riluzole 100mg compared to Control for health problem or population** | | | | | |
| --- | --- | --- | --- | --- | --- |
| **Patient or population:** Adults with MND/ALS  **Setting:** NA  **Intervention:** Riluzole 100mg  **Comparison:** Control | | | | | |
| **Outcomes** | **№ of participants**  **(studies)**  **Follow-up** | **Certainty of the evidence**  **(GRADE)** | **Relative effect**  **(95% CI)** | **Anticipated absolute effects** | |
|  |  |  |  | **Risk with Control** | **Risk difference with riluzole 100mg** |
| Mortality 12 months (<3 years) | 631  (2 RCTs) | ⨁⨁⨁◯  Moderate^a^ | **RR 0.68**  (0.54 to 0.86) | 386 per 1000 | **123 fewer per 1000**  (177 fewer to 54 fewer) |
| Adverse events (any) (<3 years) | 828  (3 RCTs) | ⨁⨁⨁◯  Moderate^b,c^ | **RR 1.02**  (0.97 to 1.08) | 731 per 1000 | **15 more per 1000**  (22 fewer to 59 more) |
| ***The risk in the intervention group** (and its 95% confidence interval) is based on the assumed risk in the comparison group and the **relative effect** of the intervention (and its 95% CI).    **CI:** confidence interval; **MD:** mean difference; **RR:** risk ratio   1. **Explanations**   ^a^ The confidence intervals have crossed a single DT, suggesting potential for large benefit (178 fewer deaths), but also potential for moderate benefit (54 fewer deaths) (downgraded one level for imprecision).  ^b^The confidence intervals have crossed multiple DTs, suggesting potential for trivial benefit or no effect (22 fewer adverse events), but also potential for small harm (59 more adverse events) (downgraded one level for imprecision).  ^c The^ study estimates fall within trivial harm or no effect (7 more adverse events) to large harm (234 more adverse events). However, statistical testing indicates this variation is consistent with random chance (I2 = 0%, P = 0.68). This inconsistency is likely contributing to the wide confidence intervals for the summary effect estimate, which cross a decision threshold (we have not downgraded for inconsistency). | | | | | |

**Supplementary 3: QUADAS-2 review-specific guidance**

**Domain 1 – Patient selection**

**Risk of bias: could the selection of patients have introduced bias?**

**Signalling question 1: was a consecutive or random sample of patients enrolled?**

We answered 'yes' if the study enrolled a consecutive or random sample of eligible patients; 'no' if the study selected patients via convenience sampling; and 'unclear' if the study did not report the method of patient selection or we could not determine it.

**Signalling question 2: did the study avoid inappropriate exclusions?**

For amyotrophic lateral sclerosis diagnosis, we answered 'yes' for studies where we did not think there were any inappropriate exclusions; we answered ‘no’ for studies where some ALS-mimicking neuromuscular disorders were excluded. We answered 'unclear' if we could not tell.

**Applicability: Are there concerns that the included patients and setting do not align with the review question?**

We are interested in how the Gold Coast criteria perform in patients clinically suspected of having ALS who were evaluated as they would be in a neurologist clinic. All the studies were conducted in an ALS referral centre; therefore, we answered 'yes' for studies.

**Domain 2 – Index test**

**Risk of bias: could the conduct or interpretation of the index test have introduced bias?**

**Signalling question 1: were the index test results interpreted without knowledge of results of the reference standard?**

We answered 'yes’ for studies which enrolled patients prospectively and 'no’ for studies which enrolled patients retrospectively.

**Signalling question 2: if a threshold was used, was it pre-specified?**

The threshold is pre-specified in all three diagnostic criteria. We answered 'yes' for all studies.

**Applicability: are there concerns that the index test, its conduct, or its interpretation differs from the review question?**

Variations in the expertise of the clinician who administers the index test may affect estimates of a test's diagnostic accuracy. We answered 'low concern' if a neurologist administered the index test.

**Domain 3 – Reference standard**

**Risk of bias: could the reference standard, its conduct, or its interpretation have introduced bias?**

**Signalling question 1: is reference standard likely to correctly classify the target condition?**

For the amyotrophic lateral sclerosis diagnosis, we answered 'yes' for studies in which the final diagnosis was based on overall judgement of the clinical course, test results, and exclusion of other diseases.

**Signalling question 2: were the reference standard results interpreted without knowledge of results of the index test?**

For this question, we answered 'yes' if the final diagnosis was made by a different clinician or at a different clinic. We also answered ‘yes’ if blinding was explicitly stated. We answered 'no' if the study stated that the reference standard result was interpreted with knowledge of the index test result. We answered 'unclear' if we could not tell.

**Applicability: are there concerns that the target condition as defined by the reference standard does not match the question?**

We answered 'yes’ for all studies as these criteria were developed specifically for diagnosis of ALS.

**Domain 4 – Flow and timing**

**Risk of bias: could the patient flow have introduced bias?**

**Signalling question 1: was there an appropriate interval between the index test and the reference standard?**

We expected to find that, for most included studies, final diagnosis was made at later follow-ups in prospectively enrolled studies. In contrast, in retrospectively enrolled studies the final diagnosis was already known at the time of administration of the index tests. However, we do not think that the patient flow would have introduced bias.

**Signalling question 2: did all patients receive the same reference standard?**

We answered 'yes' if all patients received the same reference standard for each index test; 'no' if not all patients did; and 'unclear' if we could not tell.

**Signalling question 3: were all patients included in the analysis?**

As we had access to individual participant data, we answered ‘yes’ for all studies, as we received data for all participants reported in the publications.

**Judgements for risk of bias assessments for a given domain.**

- If we answered all signalling questions for a domain 'yes', then judged risk of bias as 'low'.
- If we answered all or most signalling questions for a domain 'no', then we judged risk of bias as 'high'.
- If we answered only one signalling question for a domain 'no', we discussed further the risk of bias judgement.
- If we answered all or most signalling questions for a domain 'unclear', then we judged risk of bias as 'unclear'.
- If we answered only one signalling question for a domain 'unclear', we discussed further the risk of bias judgement.

**Supplementary 4: QUADAS-2 and QUADAS-C Judgement tables**

**Otani 2024** [11]

QUADAS

| Methodological quality | | | |
| --- | --- | --- | --- |
| Item | Authors' judgement | Risk of bias | Applicability concerns |
| DOMAIN 1: Patient selection | | | |
| Was a consecutive or random sample of patients enrolled? | Yes |  |  |
| Was a case-control design avoided? | Yes |  |  |
| Did the study avoid inappropriate exclusions? | Yes |  |  |
| Could the selection of patients have introduced bias? |  | Low Risk |  |
| Are there concerns that the included patients and setting do not match the review question? |  |  | Low Concern |
| DOMAIN 2: Index test (All studies) | | | |
| Were the index test results interpreted without knowledge of the results of the reference standard? | Unclear |  |  |
| If a threshold was used, was it pre-specified? | Yes |  |  |
| Could the conduct or interpretation of the index test have introduced bias? |  | Unclear Risk |  |
| Are there concerns that the index test, its conduct, or interpretation differ from the review question? |  |  | Low Concern |
| DOMAIN 3: Reference standard | | | |
| Is the reference standard likely to correctly classify the target condition? | Yes |  |  |
| Were the reference standard results interpreted without knowledge of the results of the index tests? | Yes |  |  |
| Reference standard does not incorporate result of index test? | Yes |  |  |
| Could the reference standard, its conduct, or its interpretation have introduced bias? |  | Low Risk |  |
| Are there concerns that the target condition as defined by the reference standard does not match the question? |  |  | Low Concern |
| DOMAIN 4: Flow and timing | | | |
| Was there an appropriate interval between index test and reference standard? | Yes |  |  |
| Did all patients receive the same reference standard? | Yes |  |  |
| Were all patients included in the analysis? | Yes |  |  |
| Could the patient flow have introduced bias? |  | Low Risk |  |

**QUADAS-C**

| Item | Authors' judgement | Risk of bias |
| --- | --- | --- |
| Was the risk of bias for each index test judged ‘low’ for this domain? | Yes |  |
| Was a fully paired or randomised design used? | Yes |  |
| Could the selection of patients have introduced bias in the comparison? |  | Low Risk |
| Was the risk of bias for each index test judged ‘low’ for this domain? | No |  |
| Were the index test results interpreted without knowledge of the results of the other index test(s)? | Yes |  |
| Is undergoing one index test unlikely to affect the performance of the other index test(s)? | Yes |  |
| Were the index tests conducted and interpreted without advantaging one of the tests? | Yes |  |
| Could the conduct or interpretation of the index tests have introduced bias in the comparison? |  | Unclear Risk |
| Was the risk of bias for each index test judged ‘low’ for this domain? | Yes |  |
| Did the reference standard avoid incorporating any of the index tests? | No |  |
| Could the reference standard, its conduct, or its interpretation have introduced bias in the comparison? |  | Low Risk |
| Was the risk of bias for each index test judged ‘low’ for this domain? | Yes |  |
| Was there an appropriate interval between the index tests? | Yes |  |
| Was the same reference standard used for all index tests? | Yes |  |
| Are the proportions and reasons for missing data similar across index tests? | Yes |  |
| Could the patient flow have introduced bias in the comparison? |  | Low Risk |

**Pugdahl 2021** [12]

QUADAS

| Methodological quality | | | |
| --- | --- | --- | --- |
| Item | Authors' judgement | Risk of bias | Applicability concerns |
| DOMAIN 1: Patient selection | | | |
| Was a consecutive or random sample of patients enrolled? | Yes |  |  |
| Was a case-control design avoided? | Yes |  |  |
| Did the study avoid inappropriate exclusions? | Yes |  |  |
| Could the selection of patients have introduced bias? |  | Low Risk |  |
| Are there concerns that the included patients and setting do not match the review question? |  |  | Low Concern |
| DOMAIN 2: Index test (All studies) | | | |
| Were the index test results interpreted without knowledge of the results of the reference standard? | Yes |  |  |
| If a threshold was used, was it pre-specified? | Yes |  |  |
| Could the conduct or interpretation of the index test have introduced bias? |  | Low Risk |  |
| Are there concerns that the index test, its conduct, or interpretation differ from the review question? |  |  | Low Concern |
| DOMAIN 3: Reference standard | | | |
| Is the reference standard likely to correctly classify the target condition? | Yes |  |  |
| Were the reference standard results interpreted without knowledge of the results of the index tests? | Yes |  |  |
| Could the reference standard, its conduct, or its interpretation have introduced bias? |  | Low Risk |  |
| Are there concerns that the target condition as defined by the reference standard does not match the question? |  |  | Low Concern |
| Reference standard does not incorporate result of index test? | Yes |  |  |
| DOMAIN 4: Flow and timing | | | |
| Was there an appropriate interval between index test and reference standard? | Yes |  |  |
| Did all patients receive the same reference standard? | Yes |  |  |
| Were all patients included in the analysis? | No |  |  |
| Could the patient flow have introduced bias? |  | Unclear Risk |  |

**QUADAS-C**

| Item | Authors' judgement | Risk of bias |
| --- | --- | --- |
| Was the risk of bias for each index test judged ‘low’ for this domain? | Yes |  |
| Was a fully paired or randomised design used? | Yes |  |
| Could the selection of patients have introduced bias in the comparison? |  | Low Risk |
| Was the risk of bias for each index test judged ‘low’ for this domain? | Yes |  |
| Were the index test results interpreted without knowledge of the results of the other index test(s)? | Yes |  |
| Is undergoing one index test unlikely to affect the performance of the other index test(s)? | Yes |  |
| Were the index tests conducted and interpreted without advantaging one of the tests? | Yes |  |
| Could the conduct or interpretation of the index tests have introduced bias in the comparison? |  | Low Risk |
| Was the risk of bias for each index test judged ‘low’ for this domain? | Yes |  |
| Did the reference standard avoid incorporating any of the index tests? | Yes |  |
| Could the reference standard, its conduct, or its interpretation have introduced bias in the comparison? |  | Low Risk |
| Was the risk of bias for each index test judged ‘low’ for this domain? | No |  |
| Was there an appropriate interval between the index tests? | Yes |  |
| Was the same reference standard used for all index tests? | Yes |  |
| Are the proportions and reasons for missing data similar across index tests? | Yes |  |
| Could the patient flow have introduced bias in the comparison? |  | Unclear Risk |

**Shen 2021**[13]

QUADAS

| Methodological quality | | | |
| --- | --- | --- | --- |
| Item | Authors' judgement | Risk of bias | Applicability concerns |
| DOMAIN 1: Patient selection | | | |
| Was a consecutive or random sample of patients enrolled? | Yes |  |  |
| Was a case-control design avoided? | Yes |  |  |
| Did the study avoid inappropriate exclusions? | No |  |  |
| Could the selection of patients have introduced bias? |  | Unclear Risk |  |
| Are there concerns that the included patients and setting do not match the review question? |  |  | Low Concern |
| DOMAIN 2: Index test (All studies) | | | |
| Were the index test results interpreted without knowledge of the results of the reference standard? | Yes |  |  |
| If a threshold was used, was it pre-specified? | Yes |  |  |
| Could the conduct or interpretation of the index test have introduced bias? |  | Low Risk |  |
| Are there concerns that the index test, its conduct, or interpretation differ from the review question? |  |  | Low Concern |
| DOMAIN 3: Reference standard | | | |
| Is the reference standard likely to correctly classify the target condition? | Yes |  |  |
| Were the reference standard results interpreted without knowledge of the results of the index tests? | Yes |  |  |
| Could the reference standard, its conduct, or its interpretation have introduced bias? |  | Low Risk |  |
| Are there concerns that the target condition as defined by the reference standard does not match the question? |  |  | Low Concern |
| DOMAIN 4: Flow and timing | | | |
| Was there an appropriate interval between index test and reference standard? | Yes |  |  |
| Did all patients receive the same reference standard? | Yes |  |  |
| Were all patients included in the analysis? | No |  |  |
| Could the patient flow have introduced bias? |  | Unclear Risk |  |

QUADAS-C

| Item | Authors' judgement | Risk of bias |
| --- | --- | --- |
| Was the risk of bias for each index test judged ‘low’ for this domain? | No |  |
| Was a fully paired or randomised design used? | Yes |  |
| Could the selection of patients have introduced bias in the comparison? |  | Unclear Risk |
| Was the risk of bias for each index test judged ‘low’ for this domain? | Yes |  |
| Were the index test results interpreted without knowledge of the results of the other index test(s)? | Yes |  |
| Is undergoing one index test unlikely to affect the performance of the other index test(s)? | Yes |  |
| Were the index tests conducted and interpreted without advantaging one of the tests? | Yes |  |
| Could the conduct or interpretation of the index tests have introduced bias in the comparison? |  | Low Risk |
| Was the risk of bias for each index test judged ‘low’ for this domain? | Yes |  |
| Did the reference standard avoid incorporating any of the index tests? | Yes |  |
| Could the reference standard, its conduct, or its interpretation have introduced bias in the comparison? |  | Low Risk |
| Was the risk of bias for each index test judged ‘low’ for this domain? | No |  |
| Was there an appropriate interval between the index tests? | Yes |  |
| Was the same reference standard used for all index tests? | Yes |  |
| Are the proportions and reasons for missing data similar across index tests? | Yes |  |
| Could the patient flow have introduced bias in the comparison? |  | Unclear Risk |

**Hannaford 2021**[14]

QUADAS

| *Methodological quality* | | | |
| --- | --- | --- | --- |
| Item | Authors' judgement | Risk of bias | Applicability concerns |
| DOMAIN 1: Patient selection | | | |
| Was a consecutive or random sample of patients enrolled? | Yes |  |  |
| Was a case-control design avoided? | Yes |  |  |
| Did the study avoid inappropriate exclusions? | Yes |  |  |
| Could the selection of patients have introduced bias? |  | Low Risk |  |
| Are there concerns that the included patients and setting do not match the review question? |  |  | Low concern |
| DOMAIN 2: Index test (All studies) | | | |
| Were the index test results interpreted without knowledge of the results of the reference standard? | Yes |  |  |
| If a threshold was used, was it pre-specified? | Yes |  |  |
| Could the conduct or interpretation of the index test have introduced bias? |  | Low Risk |  |
| Are there concerns that the index test, its conduct, or interpretation differ from the review question? |  |  | Low concern |
| DOMAIN 3: Reference standard | | | |
| Is the reference standard likely to correctly classify the target condition? | Yes |  |  |
| Were the reference standard results interpreted without knowledge of the results of the index tests? | Yes |  |  |
| Could the reference standard, its conduct, or its interpretation have introduced bias? |  | Low Risk |  |
| Are there concerns that the target condition as defined by the reference standard does not match the question? |  |  | Low concern |
| DOMAIN 4: Flow and timing | | | |
| Was there an appropriate interval between index test and reference standard? | Yes |  |  |
| Did all patients receive the same reference standard? | Yes |  |  |
| Were all patients included in the analysis? | Yes |  |  |
| Could the patient flow have introduced bias? |  | Low Risk |  |

**QUADAS-C**

| Item | Authors' judgement | Risk of bias |
| --- | --- | --- |
| Was the risk of bias for each index test judged ‘low’ for this domain? | Yes |  |
| Was a fully paired or randomised design used? | Yes |  |
| Could the selection of patients have introduced bias in the comparison? |  | Low Risk |
| Was the risk of bias for each index test judged ‘low’ for this domain? | Yes |  |
| Were the index test results interpreted without knowledge of the results of the other index test(s)? | No |  |
| Is undergoing one index test unlikely to affect the performance of the other index test(s)? | Yes |  |
| Were the index tests conducted and interpreted without advantaging one of the tests? | Yes |  |
| Could the conduct or interpretation of the index tests have introduced bias in the comparison? |  | Low Risk |
| Was the risk of bias for each index test judged ‘low’ for this domain? | Yes |  |
| Did the reference standard avoid incorporating any of the index tests? | Yes |  |
| Could the reference standard, its conduct, or its interpretation have introduced bias in the comparison? |  | Low Risk |
| Was the risk of bias for each index test judged ‘low’ for this domain? | Yes |  |
| Was there an appropriate interval between the index tests? | Yes |  |
| Was the same reference standard used for all index tests? | Yes |  |
| Are the proportions and reasons for missing data similar across index tests? | Yes |  |
| Could the patient flow have introduced bias in the comparison? |  | Low Risk |

**Ferullo 2024** [15]

QUADAS

| *Methodological quality* | | | |
| --- | --- | --- | --- |
| Item | Authors' judgement | Risk of bias | Applicability concerns |
| DOMAIN 1: Patient selection | | | |
| Was a consecutive or random sample of patients enrolled? | Yes |  |  |
| Was a case-control design avoided? | Yes |  |  |
| Did the study avoid inappropriate exclusions? | Yes |  |  |
| Could the selection of patients have introduced bias? |  | Low Risk |  |
| Are there concerns that the included patients and setting do not match the review question? |  |  | Low Concern |
| DOMAIN 2: Index test (All studies) | | | |
| Were the index test results interpreted without knowledge of the results of the reference standard? | No |  |  |
| If a threshold was used, was it pre-specified? | Yes |  |  |
| Could the conduct or interpretation of the index test have introduced bias? |  | High Risk |  |
| Are there concerns that the index test, its conduct, or interpretation differ from the review question? |  |  | Low Concern |
| DOMAIN 3: Reference standard | | | |
| Is the reference standard likely to correctly classify the target condition? | Yes |  |  |
| Were the reference standard results interpreted without knowledge of the results of the index tests? | Yes |  |  |
| Could the reference standard, its conduct, or its interpretation have introduced bias? |  | Low Risk |  |
| Are there concerns that the target condition as defined by the reference standard does not match the question? |  |  | Low Concern |
| DOMAIN 4: Flow and timing | | | |
| Was there an appropriate interval between index test and reference standard? | Yes |  |  |
| Did all patients receive the same reference standard? | Yes |  |  |
| Were all patients included in the analysis? | Yes |  |  |
| Could the patient flow have introduced bias? |  | Low Risk |  |

QUADAS-C

| Item | Authors' judgement | Risk of bias |
| --- | --- | --- |
| Was the risk of bias for each index test judged ‘low’ for this domain? | Yes |  |
| Was a fully paired or randomised design used? | Yes |  |
| Could the selection of patients have introduced bias in the comparison? |  | Low Risk |
| Was the risk of bias for each index test judged ‘low’ for this domain? | No |  |
| Were the index test results interpreted without knowledge of the results of the other index test(s)? | Yes |  |
| Is undergoing one index test unlikely to affect the performance of the other index test(s)? | Yes |  |
| Were the index tests conducted and interpreted without advantaging one of the tests? | Yes |  |
| Could the conduct or interpretation of the index tests have introduced bias in the comparison? |  | High Risk |
| Was the risk of bias for each index test judged ‘low’ for this domain? | Yes |  |
| Did the reference standard avoid incorporating any of the index tests? | Yes |  |
| Could the reference standard, its conduct, or its interpretation have introduced bias in the comparison? |  | Low Risk |
| Was the risk of bias for each index test judged ‘low’ for this domain? | Yes |  |
| Was there an appropriate interval between the index tests? | Yes |  |
| Was the same reference standard used for all index tests? | Yes |  |
| Are the proportions and reasons for missing data similar across index tests? | Yes |  |
| Could the patient flow have introduced bias in the comparison? |  | Low Risk |

**Supplementary 5: Author responses to QUADAS questions**

**Hannaford 2021**

- **Were participants enrolled consecutively or through another method (e.g., convenience sampling)?**

Consecutively recruited and entered into the database.  Data were retrospectively analysed.

- **Were any patients excluded after enrolment? If yes, what were the reasons?**

Yes, patients who did not meet the inclusion criteria were excluded.

- **Were the index test results interpreted without knowledge of the reference standard results? [Index test refers to Gold Coast, Revised El Escorial and Awaji criteria].**

Yes, the index test was interpreted without knowledge of the reference standard.

- **Were those interpreting the reference standard (Interim/final diagnosis) blinded to the index test results?**

Yes, they were blinded.

- **Were all participants included in the final analysis? If not, please clarify the reasons for exclusion**

All patients meeting the inclusion criteria were included in the final analysis—Nil exclusions as per fig 1.

**Pugdahl 2021**

- **Were participants enrolled consecutively or through another method (e.g., convenience sampling)?**

Yes

- **Were any patients excluded after enrolment? If yes, what were the reasons?**

Yes, 35 were lost to follow-up; 3 did not have MUP analysis performed (Fig. 1 in our paper)

- **Were the index test results interpreted without knowledge of the reference standard results? [Index test refers to Goldcoast, Revised El Escorial and Awaji criteria]**

Yes

- **Were those interpreting the reference standard (Interim/final diagnosis) blinded to the index test results?**

Yes

- **Were all participants included in the final analysis? If not, please clarify the reasons for exclusion**

Yes

**Shen 2021**

- **Were participants enrolled consecutively or through another method (e.g., convenience sampling)?**

Participants were enrolled consecutively in our study. All suspected ALS patients who presented to our centre during the study period were included in our registry.

- **Were any patients excluded after enrolment? If yes, what were the reasons?** 
  Yes, several patients were excluded after initial enrolment:
- 45 patients were excluded due to incomplete clinical information or because they did not attend in person.
- 13 patients were excluded after being diagnosed with non-ALS diseases (such as spinal muscular atrophy, Kennedy disease, etc.) based on laboratory tests.
- 200 patients were lost to follow-up at 6 months and therefore excluded from the final analysis.
- **Were the index test results interpreted without knowledge of the reference standard results? [Index test refers to Goldcoast, Revised El Escorial and Awaji criteria]** 
  Yes, the index test (diagnostic criteria evaluations) was interpreted by physicians who were blinded to the final (reference standard) diagnosis.
- **Were those interpreting the reference standard (Interim/final diagnosis) blinded to the index test results?** 
  Yes, the evaluators of the reference standard (final diagnosis) were blinded to the results of the index tests.
- **Were all participants included in the final analysis? If not, please clarify the reasons for exclusion.** 
  No, not all enrolled participants were included in the final analysis. As described above, participants were excluded if clinical data were incomplete, final diagnosis was a non-ALS disorder, or if they were lost to follow-up.

**Ferullo 2024**

- **Were participants enrolled consecutively or through another method (e.g., convenience sampling)?**

They were enrolled consecutively.

- **Were any patients excluded after enrollment? If yes, what were the reasons?**

No patients were excluded after enrollment.

- **Were the index test results interpreted without knowledge of the reference standard results? [Index test refers to Goldcoast, Revised El Escorial and Awaji criteria]**

Our data were collected retrospectively, so we already knew the final diagnosis. I understand this could introduce a bias, but in the materials and methods section of the paper, it is specified that the data collection was retrospective, so it wasn’t entirely blinded.

- **Were those interpreting the reference standard (Interim/final diagnosis) blinded to the index test results?**

Yes, because the final ALS diagnosis had already been established beforehand and was not influenced by the results of the diagnostic criteria we applied later during the retrospective data collection.

- **Were all participants included in the final analysis? If not, please clarify the reasons for exclusion.**

Yes, all of our participants were included in the final analysis.

**References**

1.	Jewett, G., et al., *Gold Coast criteria expand clinical trial eligibility in amyotrophic lateral sclerosis.* Muscle Nerve, 2022. **66**(4): p. 397-403.

2.	Ohnari, K., K. Mafune, and H. Adachi, *Usefulness of the Gold Coast criteria in diagnosing fast-progressing amyotrophic lateral sclerosis.* J Neurol Sci, 2025. **471**: p. 123418.

3.	de Jongh, A.D., et al., *Characterising ALS disease progression according to El Escorial and Gold Coast criteria.* J Neurol Neurosurg Psychiatry, 2022. **93**(8): p. 865-870.

4.	Desmaison, A., et al., *Upper motor neuron assessment in amyotrophic lateral sclerosis using the patellar tendon reflex and motor-evoked potentials to the lower limbs.* Rev Neurol (Paris), 2024. **180**(7): p. 632-641.

5.	Laucius, O., et al., *Ultrasonography of the Vagus Nerve for ALS Patients: Correlations with Clinical Data and Dysfunction of the Autonomic Nervous System.* Medicina (Kaunas), 2025. **61**(5).

6.	Vucic, S., et al., *Gold Coast diagnostic criteria: Implications for ALS diagnosis and clinical trial enrollment.* Muscle Nerve, 2021. **64**(5): p. 532-537.

7.	Ortiz-Corredor, F., et al., *Profiles of disease progression and predictors of mortality in Colombian patients with amyotrophic lateral sclerosis: a comprehensive longitudinal study.* Amyotroph Lateral Scler Frontotemporal Degener, 2025. **26**(1-2): p. 141-148.

8.	Stikvoort García, D.J.L., et al., *Diagnostic accuracy of nerve excitability and compound muscle action potential scan derived biomarkers in amyotrophic lateral sclerosis.* Eur J Neurol, 2023. **30**(10): p. 3068-3078.

9.	Stern, C., et al., *Protocol for the Development of The Australian Motor Neurone Disease (MND) Guideline.* Clinical and Public Health Guidelines, 2026. **3**(1): p. e70055.

10.	Richards, D., J.A. Morren, and E.P. Pioro, *Time to diagnosis and factors affecting diagnostic delay in amyotrophic lateral sclerosis.* Journal of the Neurological Sciences, 2020. **417**: p. 117054.

11.	Otani, R., et al., *Diagnostic utility of Gold Coast criteria for amyotrophic lateral sclerosis in Asia.* Amyotroph Lateral Scler Frontotemporal Degener, 2024. **25**(3-4): p. 264-270.

12.	Pugdahl, K., et al., *Gold Coast diagnostic criteria increase sensitivity in amyotrophic lateral sclerosis.* Clin Neurophysiol, 2021. **132**(12): p. 3183-3189.

13.	Shen, D., et al., *The Gold Coast criteria increases the diagnostic sensitivity for amyotrophic lateral sclerosis in a Chinese population.* Transl Neurodegener, 2021. **10**(1): p. 28.

14.	Hannaford, A., et al., *Diagnostic Utility of Gold Coast Criteria in Amyotrophic Lateral Sclerosis.* Ann Neurol, 2021. **89**(5): p. 979-986.

15.	Ferullo, L., et al., *Gold Coast Criteria in ALS Diagnosis: A Real-World Experience.* Brain Sci, 2024. **14**(11).
